# Transcriptomic analysis reveals specific metabolic pathways of enterohemorrhagic *Escherichia coli* O157:H7 in bovine digestive contents

**DOI:** 10.1186/s12864-018-5167-y

**Published:** 2018-10-23

**Authors:** Audrey Segura, Marine Bertoni, Pauline Auffret, Christophe Klopp, Olivier Bouchez, Clémence Genthon, Alexandra Durand, Yolande Bertin, Evelyne Forano

**Affiliations:** 1 Université Clermont Auvergne, INRA, UMR 0454 MEDIS, F-63000 Clermont-Ferrand, France; 2 Plateforme Bioinformatique Toulouse, Midi-Pyrénées UBIA, INRA, Auzeville, Castanet-Tolosan, France; 3 INRA, US 1426, GeT-PlaGe, Genotoul, Castanet-Tolosan, France; 4 Present address : Institut National de Police Scientifique – Laboratoire de Police Scientifique de Marseille, Marseille, France; 5 Present address : Ifremer, UMR 241 EIO, Tahiti, French Polynesia

**Keywords:** EHEC O157:H7, Bovine gastrointestinal tract, RNA-seq, Metabolism, Carbon sources, Mucus-derived carbohydrates, Ethanolamine, Respiration

## Abstract

**Background:**

The cattle gastrointestinal tract (GIT) is the main enterohemorrhagic *Escherichia coli* (EHEC) reservoir. In order to identify nutrients required for the survival or multiplication of EHEC in the bovine GIT, we compared the transcriptomes of the EHEC O157:H7 reference strain EDL933 cultured *in vitro* in bovine digestive contents (DCs) (rumen, small intestine and rectum) using RNA-sequencing.

**Results:**

Gene expression profiles showed that EHEC EDL933 activated common but also specific metabolic pathways to survive in the different bovine DCs. Mucus-derived carbohydrates seem important in EHEC nutrition in posterior DCs (small intestine and rectum) but not in rumen content. Additional carbohydrates (xylose, ribose, mannitol, galactitol) as well as gluconeogenic substrates (aspartate, serine, glycerol) would also be used by EHEC as carbon and/or nitrogen sources all along the bovine GIT including the rumen. However, xylose, GalNac, ribose and fucose transport and/or assimilation encoding genes were over-expressed during incubation in rectum content compared with rumen and intestine contents, and genes coding for maltose transport were only induced in rectum. This suggests a role for these carbohydrates in the colonization of the cattle rectum, considered as the major site for EHEC multiplication. In contrast, the transcription of the genes associated with the assimilation of ethanolamine, an important nitrogen source for EHEC, was poorly induced in EHEC growing in rectum content, suggesting that ethanolamine is mainly assimilated in the cattle rumen and small intestine. Respiratory flexibility would also be required for EHEC survival because of the redundancy of dehydrogenases and reductases simultaneously induced in the bovine DCs, probably in response to the availability of electron donors and acceptors.

**Conclusion:**

EHEC EDL933 showed a high flexibility in the activation of genes involved in respiratory pathways and assimilation of carbon and nitrogen sources, most of them from animal origin. This may allow the bacterium to adapt and survive in the various bovine GIT compartments. Obtaining a better understanding of EHEC physiology in bovine GIT is a key step to ultimately propose strategies to limit EHEC carriage and shedding by cattle.

**Electronic supplementary material:**

The online version of this article (10.1186/s12864-018-5167-y) contains supplementary material, which is available to authorized users.

## Background

Enterohemorrhagic *Escherichia coli* (EHEC) are Shiga toxin-producing *E. coli* (STEC) which represent a well-known group of foodborne zoonotic pathogens distributed worldwide. Their ability to produce Shiga toxins in human gut constitutes their main virulence attribute and leads to diseases ranging from uncomplicated diarrhea to hemorrhagic colitis (HC) and hemolytic and uremic syndrome (HUS), the principal cause of kidney failure in children [1]. Numerous outbreaks and sporadic cases of severe HC and HUS have been attributed to the O157:H7 serotype [2]. Epidemiological studies indicate that the healthy bovine gastrointestinal tract (GIT) is the main reservoir of EHEC O157:H7 [3]. Bovines are asymptomatic carriers because they lack globotriaosylceramide, the Shiga toxin receptor necessary for intestinal and renal endothelium pathophysiological effects [4, 5]. Bovine feces excreted in the environment are the principal source of herd contamination and human infection. Human infection is typically acquired through the consumption of contaminated food (undercooked meat, dairy products, fruits and vegetables) and water. On farm strategies to decrease EHEC carriage by cattle should efficiently reduce shedding and consequently human EHEC infection incidence [6]. Understanding of EHEC physiology during its transit through the bovine GIT and identifying nutrients preferentially used by EHEC to survive in the bovine gut are critical to propose nutritional strategies limiting EHEC shedding.

In the rumen, EHEC O157:H7 are able to survive but not grow, probably due to inhibition by the endogenous microbiota and/or physicochemical conditions [7–9]. Indeed, the rumen is not considered as an EHEC colonization site. However, the pathogen must be able to survive in the rumen, to cross the acidic barrier of the abomasum and to transit to the posterior digestive compartments, constituting more favorable environments for EHEC growth. *E. coli* O157 have been detected throughout the gut of experimentally inoculated or naturally infected animals even if the rectum is considered the main EHEC colonization site [10–15].

Little is known about the nutrients used and the metabolic pathways required for EHEC survival and/or multiplication in the bovine GIT. The GIT content is a complex ecosystem composed of a dense and diverse microbiota, and EHEC survival relies on competition for a variety of energy, carbon and nitrogen sources. According to the Freter niche theory, each bacterial species must use one or several nutrients more efficiently than other species to colonize the mammalian intestine [16]. In this complex ecosystem, available nutrients derive from the animal diet as well as eukaryotic cell secretion and debris released in the luminal content after eukaryotic and prokaryotic cell apoptosis. Such nutrients can be of different composition and are present at various concentrations depending on the intestinal site. We previously showed that carbohydrates released by the mucus layer covering the small intestine enterocytes (mannose, N-acetylglucosamine [GlcNAc], N-acetylneuraminic acid [Neu5Ac] and galactose) were used by EHEC to grow in bovine small intestine content [17]. Furthermore, each monosaccharide was used more rapidly by EHEC than by the endogenous microbiota, suggesting an effective assimilation of mucus-derived carbohydrates by the pathogen [17]. In addition, previous studies showed that i) the genes *dctA* and *agaB*, encoding the specific transport of C_4_-dicarboxylates and N-acetylgalactosamine (GalNac), respectively, are involved in EHEC colonization in the calf intestine and ii) fucose is an important carbon source for maintenance of EHEC in the cattle rectum [18, 19]. Also, ethanolamine (EA), released in the mammal gut by animal, plant and bacterial cell membrane phospholipids, is an important nitrogen source giving a competitive growth advantage to EHEC in bovine small intestine content [20]. Furthermore, the gluconeogenesis pathway is also involved in the survival and maintenance of EHEC in bovine small intestine content and several compounds such as glycerol, lactate and amino acids were identified as gluconeogenic substrates present in the bovine small intestine [21]. However, the presence of nutrients and their assimilation by EHEC in other digestive compartments are poorly documented.

The objective of this study was to identify nutrients and metabolic pathways potentially used by EHEC in different bovine digestive compartments, from rumen to rectum, in order to improve our knowledge of EHEC physiology in the animal GIT. We compared the transcriptomic profiles of an EHEC O157:H7 reference strain grown *in vitro* in rumen, small intestine and rectum contents and in minimal medium by the RNA-sequencing (RNA-seq) approach. Quantification of differentially expressed transcripts represents a method of choice to reveal bacterial transcriptomes in complex environments such as the mammalian intestine. Our results highlighted that EDL933 is well-adapted to the bovine gut environment and activates common but also specific metabolic pathways to survive in the contents of different bovine digestive compartments.

## Results

### Growth and survival of EHEC EDL933 in bovine digestive contents

**Fig. 1:**
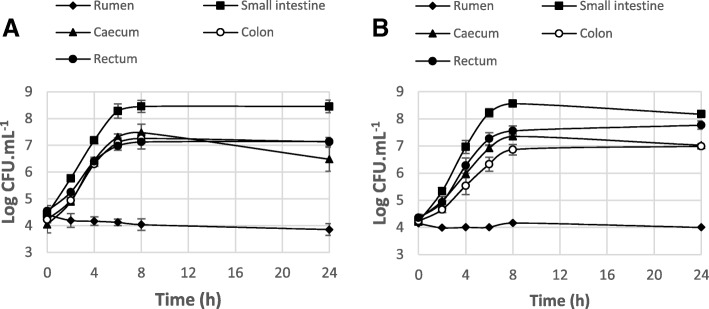
Growth curves of EDL933 Rif^R^ incubated in bovine digestive contents. (**a)** Unfiltered DCs; (**b**) Filtered DCs. CFU enumeration at each time point is the mean of at least three independent experiments. The presented values are the log_10_ mean number of CFU mL^-1^ ± the standard error of the mean (SEM). Statistical analyses were performed using the two-way ANOVA with the Bonferroni post-hoc test. In unfiltered DCs, growth yield in small intestine content was significantly different from that in the other DCs (*p* < 0.001); growth yield in caecum, colon and rectum contents was significantly different from that in rumen and small intestine contents (*p* < 0.001). In filtered DCs, growth yield in all DCs was significantly different from that in rumen content (*p* < 0.001); growth yield in small intestine content was significantly different from that in caecum and colon contents (*p* < 0.001); growth yield in rectum content was significantly different from that in caecum and colon contents (*p* < 0.05)

The culture conditions of *E. coli* strains in bovine digestive contents (DCs) (rumen, small intestine, caecum, colon and rectum contents) were chosen to mimic the physiological environment of each compartment (see the Materials and Methods section). Bacterial growth of EDL933 Rif^R^ was analyzed in each DC and compared to the growth patterns of the spontaneous Rif^R^ mutants of the strains Sakai (O157:H7 EHEC), NV95 (bovine O157:H7 STEC) and BG1 (bovine commensal *E. coli*) (Fig. 1) (Additional file 1: Table S1). As shown in Fig. 1a, EDL933 Rif^R^ was able to grow in all DCs, except in rumen content. In the rumen, a ≈ 0.5 log CFU mL^-1^ decrease in the EDL933 Rif^R^ population was observed after 8h of incubation and the strain survived until 24h at a low level (< 10^4^ CFU mL^-1^). In contrast, EDL933 Rif^R^ grew with a high yield (≈ 4 log CFU mL^-1^ increase) in small intestine content and remained at high concentration up to 24h of incubation. In caecum, colon and rectum contents, the EDL933 Rif^R^ population increased from ≈ 4 to ≈ 7 log CFU mL^-1^ and then remained stable up to 24h of incubation. Similar growth patterns were obtained with the other *E. coli* strains incubated in each DC (no statistically significant difference was observed between the strains tested) (Additional file 1: Figure S2).

Short chain fatty acid (SCFA) concentration and pH were measured before and after incubation in the DC. In rumen content, the pH became more acidic than in the other DCs and decreased about 1.4 pH units after 24h of incubation, indicating a fermentative activity of the endogenous ruminal microbiota (Table 1). In the other DCs, pH values were all between 7 and 8 whatever the incubation time (Table 1) which corresponded to more favorable conditions for *E. coli* multiplication. Before incubation, the total SCFA concentration was higher in rumen content than in the other DC’s (Table 1) with a high acetate concentration (84.51 ± 6.93 mM in rumen content, result not shown). Otherwise, total SCFA concentration increased in all DCs after 24h of incubation, indicating active endogenous microbiota (Table 1).

**Table 1:** pH and SCFA concentrations in unfiltered bovine digestive contents before incubation (t=0) and after 6h and 24h of EHEC EDL933 incubation

	Time	Rumen	Small intestine	Caecum^a^	Colon^a^	Rectum^a^
pH	0	7.25 (± 0.05)	7.22 (± 0.01)	7.29 (± 0.23)	7.42 (± 0.39)	7.38 (± 0.42)
	6h	6.30 (± 0.04)	7.14 (± 0.02)	7.90 (± 0.20)	7.53 (± 0.03)	7.46 (± 0.00)
	24h	5.85 (± 0.02)	7.66 (± 0.02)	7.88 (± 0.33)	7.38 (± 0.17)	7.59 (± 0.06)
Total SCFAs (mM)	0	115.78 (± 9.56)	15.75 (± 0.58)	36.27 (± 0.69)	38.49 (± 2.28)	19.35 (± 1.82)
	6h	90.80^b^	31.94^b^	27.66^b^	37.36^b^	25.01 (± 0.33)
	24h	129.53^b^	50.31^b^	37.01^b^	70.61^b^	50.21 (± 2.35)

The presented values are the mean of at least three measures (± SEM).

^a^pH and SCFA concentrations were measured in the incubation media (i.e. after dilution of DC for those which were diluted, see materials and methods)

^b^SCFA concentrations were measured only once for these samples

EHEC EDL933 growth was also monitored in the absence of endogenous microbiota (filtered DC, Fig. 1b). The growth rate and yield of the bacteria grown in filtered or unfiltered DC (Fig. 1a and b) were similar except a slightly but not statistically significant lower rate in filtered colon content. This suggests that the lack of growth of EDL933 in rumen content is due to the physicochemical conditions (more acidic pH and higher SCFA concentration) and/or the presence of inhibitory compounds in rumen fluids rather than nutritional competition with the endogenous microbiota.

### Transcriptome profiling of EHEC EDL933 incubated in filtered bovine digestive contents

To provide insight about nutrients and metabolic pathways preferentially used by EHEC to survive in the bovine gut, we performed RNA-seq analysis to compare global gene expression changes in EHEC EDL933 grown in filtered DCs (rumen, small intestine and rectum contents) and in M9 minimal medium supplemented with glucose as the sole carbon source (M9-Glc). Note that these DCs were chosen because (i) the rumen is the first digestive compartment encountered by EHEC during their transit, (ii) the small intestine is the central compartment in intestinal absorption and (iii) the rectum is described as the primary site of EHEC colonization and multiplication and is the last compartment before fecal excretion. M9-Glc was chosen as a reference medium because i) it was used as such in previous transcriptomic studies in our laboratory [17, 20, 21] and ii) M9-Glc is a completely defined medium, with glucose as sole carbon source; iii) glucose concentration is very low in the bovine GIT [17].

**Fig. 2:**
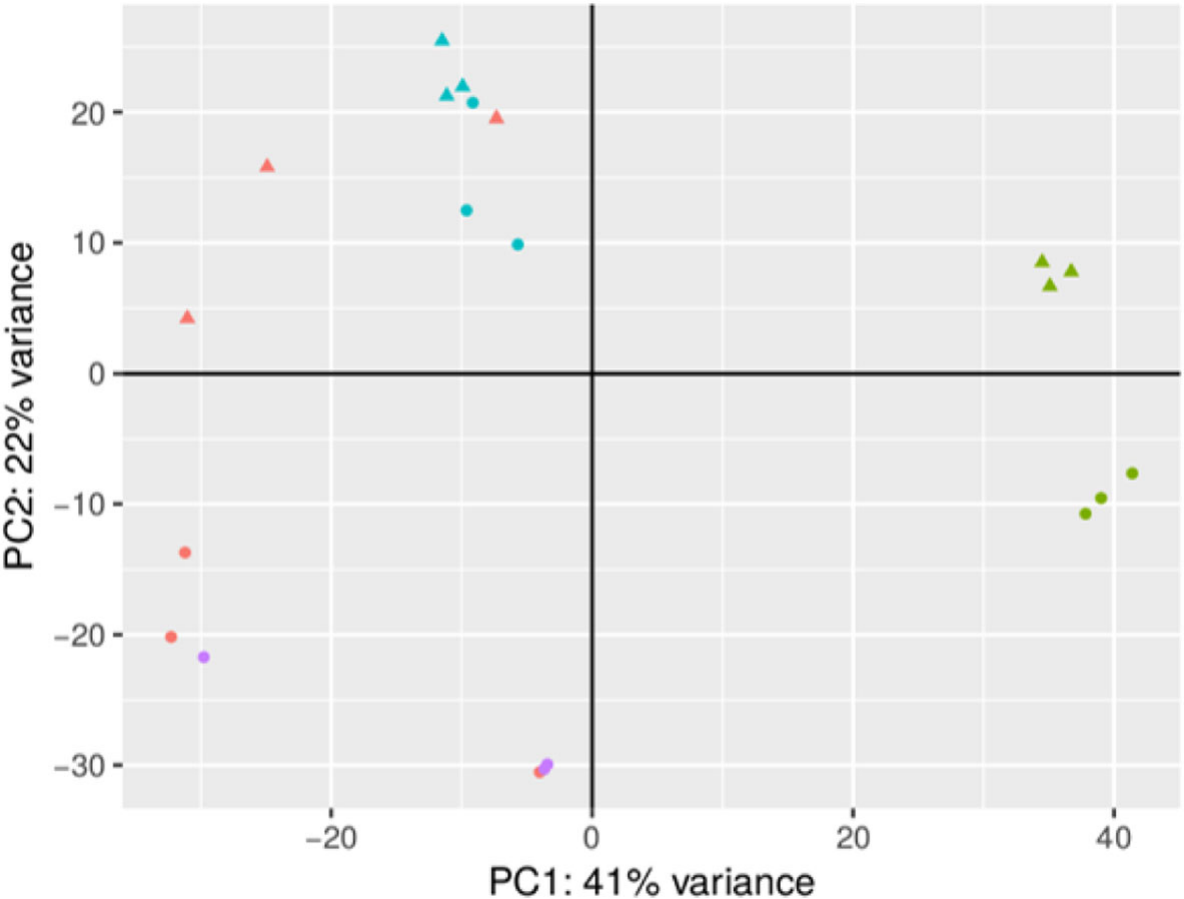
Principal component analysis results for the transcriptomes of EDL933 grown in digestive contents. Transcriptomes of EDL933 incubated in rumen content during 6h (purple circles), and in small intestine content (red), rectum content (blue) and M9-Glc (green) at the mid-exponential (triangle) and the late-exponential (circle) growth phases. Three biological replicates were analyzed for each condition

Transcriptomic profiles were determined from mRNA samples collected during mid- and late-exponential growth phases (3h and 6h of incubation, respectively), except for rumen content in which RNA samples were collected only after 6h of incubation (Additional file 1: Figure S1). The whole transcriptomes of EDL933 grown in the different DCs were first analyzed by a principal component analysis. The first principal component (PC1, accounting for 41% of the variation) and the second principal component (PC2, accounting for 22% of the variation) together explained 63% of the variance in the RNA-Seq data (Fig. 2). The transcriptomes of EDL933 grown in M9-Glc and harvested at the different growth phases formed two clusters which were distant from the other transcriptomes. In addition, the global gene expression profiles of EDL933 grown in rumen and rectum contents also showed different patterns.

Statistical analysis revealed that from 540 to 701 genes carried by the EDL933 genome were differentially expressed (log2 fold-change [log2FC] > │2│; *q*-value < 0.05) in the three DCs compared to M9-Glc (Table 2). We also compared the number of genes up and down-regulated between the digestive contents (Table 2). The highest differences were found between rumen and rectum contents after 6h incubation (678 genes differentially expressed). To validate the data, the expression level of 32 genes found to be differentially expressed using RNA-Seq measures was quantified by RT-qPCR on samples collected under all experimental conditions. The results confirmed alteration of the targeted gene transcription in the different DC relative to M9-Glc (Additional file 1: Table S4). The sets of genes up and down-regulated in the three different DCs relative to M9-Glc are presented in Additional file 2: Table S5.

**Table 2:** Number of differentially expressed genes (log2 FC > │2│; *q* < 0.05) in EHEC EDL933 incubated in filtered digestive contents and M9-Glc

Digestive contents	up^a^	down^b^	Total
After 3 hours of incubation			
Small intestine/M9	347	354	701
Rectum/M9	290	250	540
Small intestine/rectum	109	171	280
After 6 hours of incubation			
Rumen/M9	337	280	617
Small intestine/M9	346	254	600
Rectum/M9	305	320	625
Rectum/rumen	277	401	678
Small intestine/rectum	283	173	456

^a^number of up-regulated genes ; ^b^number of down-regulated genes

The comparison between small intestine and rumen after 6h of incubation does not appear in the table because of the low significance of the statistical analysis

To facilitate subsequent analyses, the differentially expressed genes were classified into cluster of orthologous groups (COGs) in order to assign a physiological function to each gene. Note that some genes were classified in more than one category. The results showed that 36.4% of the differentially expressed genes in EDL933 grown in the three DCs were found within the functional categories “Carbohydrate transport and metabolism” (n=333), “Amino acid transport and metabolism” (n=467) and “Energy production and conversion” (n=321) (Additional file 3: Table S6). Genes of unknown and unclassified functions represented 22.9% of the genes differentially expressed in the three DCs (Additional file 3: Table S6). Most of the other functional categories contained less than 10% of differentially expressed genes (Additional file 3: Table S6).

In depth analysis of the genes commonly or specifically up-regulated in EHEC EDL933 incubated in the bovine DCs and included in the main functional categories described above was conducted in order to predict i) the metabolic pathways used and compounds assimilated by EHEC in bovine DCs and ii) the sequential nutrition of EHEC during incubation in the bovine DCs.

### Genes up-regulated relative to carbon sources

#### Carbon sources common to the rumen, small intestine and rectum contents

**Fig. 3:**
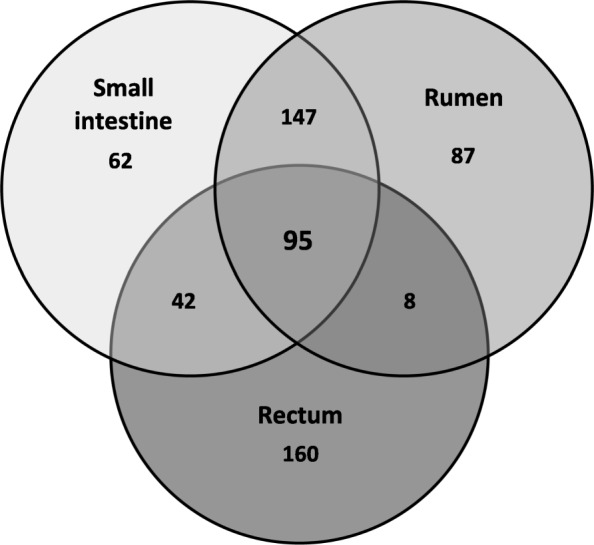
Venn diagram of up-regulated genes in EHEC EDL933 incubated in bovine DCs. The cells were collected after 6h of incubation in the three DCs compared to M9-Glc

**Fig. 4:**
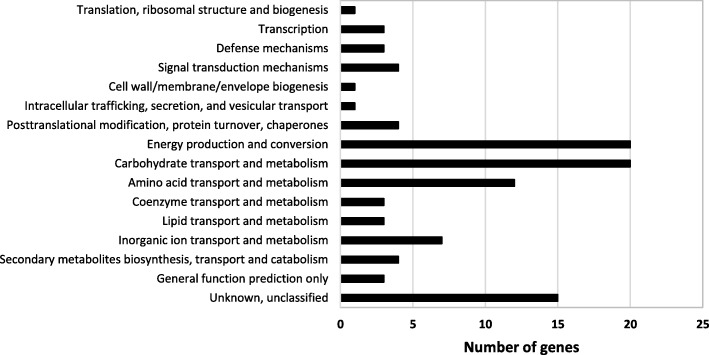
Functional classification of the 95 genes commonly up-regulated in EDL933 after 6h of incubation in filtered bovine DCs relative to M9-Glc

We first analyzed the genes commonly up-regulated in EHEC EDL933 incubated in the three DCs compared to M9-Glc. A Venn diagram (Fig. 3) shows that 95 genes were commonly up-regulated in EDL933 incubated in the three DCs after 6h of incubation. As shown in Fig. 4 and Table S7 (Additional file 3), 52 of the 95 genes (54.7%) were associated with the three main functional categories mentioned above. Expression of the genes coding for proteins involved in the transport and/or metabolism of carbon sources (carbohydrates, amino acids, glycerol, and C_4_-dicarboxylates) (Additional file 3: Table S7) were subsequently analyzed.

The genes *xylF* and *xylA* (transport and catabolism of xylose, respectively), *fucA* and *fucO* (fucose catabolism) and *rbsACD* (ribose transporter) were commonly up-regulated in EDL933 grown in the three DCs (Table 3). The genes *agaEVW* and *agaYZ* encoding the transport and catabolism of N-acetylgalactosamine (GalNAc), respectively, as well as the genes *cmtB* (mannitol transporter) and *z4875* and *z4876* (galactitol transporter) also exhibited increased expression (Table 3). Note that most of these genes (except *agaY*, *rbsACD*, *z4875* and *z4876*) were expressed at higher levels in EDL933 incubated in small intestine and rectum contents than in rumen content (Table 3).

**Table 3:** RNA-Seq data

Gene tag	Gene	Gene product	3 hours incubation				6 hours incubation					
			Small intestine		Rectum		Small intestine		Rectum		Rumen	
			Log2FC	*q*-value	Log2FC	*q*-value	Log2FC	*q*-value	Log2FC	*q*-value	Log2FC	*q*-value
Z2315	*acpD*	FMN-dependent NADH-azoreductase	2.53	7.91E-05	5.12	7.34E-17	3.16	6.19E-07	4.77	7.25E-15	2.38	2.65E-04
Z4492	*agaB*	PTS system galactosamine-specific IIB component	NDE		3.01	2.87E-05	NDE		3.27	2.44E-06	NDE	
Z4493	*agaC*	PTS system galactosamine-specific IIC component	NDE		2.11	4.41E-03	NDE		2.15	1.61E-03	NDE	
Z4487	*agaE*	PTS system N-acetylgalactosamine-specific IID component	NDE		3.97	1.62E-10	2.60	4.74E-05	3.99	6.14E-11	2.44	1.35E-04
Z4488	*agaF*	PTS system N-acetylgalactosamine-specific IIA component	NDE		3.23	1.41E-08	NDE		3.20	7.02E-09	NDE	
Z4485	*agaV*	PTS system N-acetylgalactosamine-specific IIB component	NDE		6.10	6.41E-23	3.22	5.89E-07	4.79	1.45E-14	2.52	1.40E-04
Z4486	*agaW*	PTS system N-acetylgalactosamine-specific IIC component	NDE		5.36	7.49E-22	3.37	5.24E-09	5.06	1.66E-19	3.07	1.25E-07
Z4491	*agaY*	Tagatose 1,6-diphosphate aldolase (GatY/KbaY subunit)	NDE		4.57	1.82E-13	2.54	7.96E-05	3.85	4.58E-10	2.69	2.25E-05
Z4484	*agaZ*	Tagatose 1,6-diphosphate aldolase (GatZ/KbaZ subunit)	NDE		5.81	2.92E-18	2.54	4.57E-04	4.24	5.68E-10	2.30	1.52E-03
Z1396	*appB*	Cytochrome bd oxidase subunit II	NDE		NDE		3.90	6.57E-08	NDE		3.18	1.60E-05
Z1395	*appC*	Cytochrome bd ubiquinol oxidase subunit I	NDE		NDE		4.33	7.02E-09	NDE		3.60	2.31E-06
Z0070	*araA*	L-arabinose isomerase	2.79	2.22E-03	NDE		3.14	6.37E-04	2.22	1.88E-02	NDE	
Z0072	*araB*	L-ribulokinase	3.80	7.49E-05	3.49	4.33E-04	3.65	1.74E-04	3.82	7.55E-05	NDE	
Z0069	*araD*	L-ribulose 5-phosphate 4-epimerase	2.81	1.03E-03	NDE		2.26	1.19E-02	NDE		NDE	
Z2954	*araF*	L-arabinose transport system (substrate-binding protein)	4.06	1.61E-05	2.69	8.44E-03	4.56	1.30E-06	4.13	1.41E-05	NDE	
Z2953	*araG*	L-arabinose transport system (ATP-binding protein)	2.57	6.40E-03	NDE		3.08	1.08E-03	2.09	3.18E-02	NDE	
Z5245	*asnA*	Aspartate ammonia-ligase	NDE		2.68	9.66E-04	3.11	8.23E-05	2.84	3.50E-04	3.05	1.14E-04
Z0821	*asnB*	Asparagine synthase	NDE		2.84	2.54E-04	NDE		NDE		NDE	
Z5744	*aspA*	Aspartate ammonia-lyase	6.08	3.44E-23	5.03	8.65E-16	5.34	5.67E-18	2.84	1.33E-05	4.46	1.04E-12
Z5734	*cadA*	Lysine decarboxylase	NDE		NDE		2.81	1.61E-03	NDE		3.33	1.21E-04
Z5735	*cadB*	Cadaverine:lysine antiporter	NDE		NDE		3.96	2.74E-05	NDE		4.15	9.05E-06
Z3712	*cchB*	Ethanolamine utilization protein EutN	NDE		NDE		4.3	4.58E-04	NDE		4.47	2.33E-04
Z0762	*citC*	[citrate (pro-3S)-lyase] ligase	NDE		NDE		NDE		NDE		2.02	8.56E-03
Z4278	*cmtB*	Mannitol-specific IIA component	2.35	4.79E-03	3.04	2.28E-04	2.10	1.38E-02	3.02	1.74E-04	2.04	1.38E-02
Z0901	*cydB*	Cytochrome bd ubiquinol oxydase (subunit II)	2.12	1.29E-04	2.37	2.32E-05	NDE		NDE		NDE	
Z4942	*dctA*	Aerobic C_4_-dicarboxylates transporter	4.13	1.21E-09	3.91	1.73E-08	3.35	1.60E-06	3.65	1.29E-07	2.11	3.99E-03
Z5743	*dcuA*	Anaerobic C_4_-dicarboxylates transporter	3.21	2.46E-08	2.51	2.73E-05	2.12	5.02E-04	NDE		NDE	
Z5725	*dcuB*	Anaerobic C_4_-dicarboxylates transporter	2.98	2.64E-05	3.49	8.54E-07	3.26	4.39E-06	NDE		2.11	4.48E-03
Z1240	*dmsA*	Dimethyl sulfoxide reductase subunit A	3.01	3.54E-07	NDE		3.47	3.43E-09	NDE		3.07	2.25E-07
Z1241	*dmsB*	Dimethyl sulfoxide reductase subunit B	3.48	6.54E-08	NDE		3.56	3.63E-08	NDE		2.97	6.04E-06
Z1242	*dmsC*	Dimethyl sulfoxide reductase subunit C	2.56	3.33E-05	NDE		2.59	3.36E-05	NDE		2.29	2.76E-04
Z3707	*eutA*	Ethanolamine utilization protein EutA	NDE		NDE		3.52	2.76E-03	2.75	2.10E-02	4.04	4.15E-04
Z3706	*eutB*	Ethanolamine ammonia-lyase (large subunit)	NDE		NDE		3.68	1.68E-03	3.12	8.17E-03	4.02	4.60E-04
Z3705	*eutC*	Ethanolamine ammonia-lyase (small subunit)	NDE		NDE		3.37	4.18E-03	2.79	1.85E-02	3.62	1.71E-03
Z3711	*eutE*	Aldehyde dehydrogenase	NDE		NDE		4.14	7.46E-04	NDE		4.50	2.01E-04
Z3709	*eutG*	Alcohol dehydrogenase	NDE		NDE		3.87	1.59E-03	NDE		4.42	2.15E-04
Z3708	*eutH*	Ethanolamine transporter	NDE		NDE		3.42	3.79E-03	NDE		4.10	3.45E-04
Z3714	*eutI*	Phosphotransacetylase	NDE		NDE		4.19	4.95E-04	NDE		4.51	1.43E-04
Z3710	*eutJ*	Ethanolamine utilization protein EutJ	NDE		NDE		3.64	3.76E-03	NDE		4.25	4.99E-04
Z3703	*eutK*	Ethanolamine utilization protein EutK	NDE		NDE		3.22	6.41E-03	3.01	1.02E-02	3.52	2.29E-03
Z3704	*eutL*	Ethanolamine utilization protein EutL	NDE		NDE		3.28	5.36E-03	2.82	1.70E-02	3.51	2.40E-03
Z3713	*eutM*	Ethanolamine utilization protein EutM	NDE		NDE		4.37	2.51E-04	NDE		4.64	8.61E-05
Z3717	*eutP*	Ethanolamine utilization protein EutP	NDE		NDE		3.80	1.76E-03	2.69	3.04E-02	4.33	2.60E-04
Z3716	*eutQ*	Ethanolamine utilization protein EutQ	NDE		NDE		3.87	1.56E-03	NDE		4.25	3.92E-04
Z3718	*eutS*	Ethanolamine utilization protein EutS	NDE		NDE		3.63	2.93E-03	3.27	7.36E-03	4.54	1.17E-04
Z2236	*fdnG*	Formate dehydrogenase-N (α subunit)	NDE		NDE		2.73	6.18E-06	3.48	3.63E-09	2.06	9.55E-04
Z2235	*fdnH*	Formate dehydrogenase-N (β subunit)	3.63	5.60E-08	NDE		NDE		NDE		NDE	
Z2234	*fdnI*	Formate dehydrogenase-N (γ subunit)	3.02	2.25E-09	NDE		NDE		NDE		NDE	
Z5435	*fdoH*	Formate dehydrogenase-I (iron-sulfur subunit)	NDE		NDE		3.2	2.40E-06	3.92	3.75E-09	2.51	3.15E-04
Z5434	*fdoI*	Formate dehydrogenase-I (γ subunit)	NDE		NDE		2.84	2.29E-05	3.53	6.94E-08	2.11	2.25E-03
Z5762	*frdA*	Fumarate reductase (flavoprotein subunit)	4.43	1.26E-12	2.24	9.63E-04	4.70	4.78E-14	2.02	3.04E-03	4.03	1.64E-10
Z5760	*frdB*	Fumarate reductase (iron-sulfur subunit)	4.90	4.39E-12	2.28	3.74E-03	4.88	6.63E-12	NDE		4.17	7.12E-09
Z5759	*frdC*	Fumarate reductase (C subunit)	4.92	3.51E-11	2.27	6.14E-03	4.57	1.13E-09	NDE		3.83	5.31E-07
Z5758	*frdD*	Fumarate reductase (D subunit)	4.85	1.93E-10	2.51	2.61E-03	4.04	1.93E-07	NDE		3.20	5.72E-05
Z3425	*fruA*	PTS system fructose-specific IIB component	2.95	3.71E-04	2.44	5.39E-03	NDE		NDE		NDE	
Z3427	*fruB*	PTS system fructose-specific IIA component	3.24	4.31E-04	2.67	6.21E-03	2.59	7.71E-03	NDE		NDE	
Z3426	*fruK*	1-phosphofructokinase	3.38	1.07E-04	2.76	2.79E-03	2.12	2.73E-02	NDE		NDE	
Z4117	*fucA*	L-fuculose-phosphate aldolase	3.99	4.76E-07	4.06	4.41E-07	3.45	1.86E-05	4.24	7.00E-08	2.69	1.14E-03
Z4119	*fucI*	L-fucose/D-arabinose isomerase	4.12	5.83E-08	4.47	5.45E-09	2.87	3.07E-04	5.40	4.28E-13	NDE	
Z4120	*fucK*	L-fuculokinase	2.50	3.90E-04	2.37	1.16E-03	NDE		3.34	1.32E-06	NDE	
Z4116	*fucO*	Lactaldehyde reductase	3.22	1.17E-08	3.36	6.44E-06	3.39	4.25E-06	3.92	6.72E-08	2.91	9.95E-05
Z4118	*fucP*	L-fucose permease	3.62	5.13E-07	4.44	7.68E-10	2.62	5.68E-04	5.13	3.02E-13	NDE	
Z4121	*fucU*	L-fucose mutarotase	NDE		NDE		NDE		2.05	1.07E-03	NDE	
Z2615	*fumA*	Fumarase	3.90	5.07E-19	3.35	7.45E-14	3.50	2.06E-15	3.53	1.57E-15	2.50	3.54E-08
Z5724	*fumB*	Fumarate hydratase class I	3.57	1.16E-05	3.72	6.56E-06	3.76	4.38E-06	NDE		2.68	1.56E-03
Z2614	*fumC*	Fumarate hydratase class II	2.99	4.63E-10	2.19	1.31E-05	2.46	4.60E-07	NDE		NDE	
Z0462	*fusK*	Fucose sensing two-component system (sensor)	NDE		3.12	3.86E-17	NDE		2.45	5.35E-11	NDE	
Z0463	*fusR*	Fucose sensing two-component system (regulator)	3.37	2.15E-09	3.81	1.78E-11	NDE		3.58	2.40E-10	NDE	
Z0927	*galK*	Galactokinase	2.55	1.80E-05	3.50	2.71E-09	NDE		NDE		NDE	
Z0926	*galM*	Aldolase-1-epimerase	2.04	6.59E-04	2.62	1.02E-05	NDE		NDE		NDE	
Z4288	*galP*	MFS transporter galactose:H^+^ symporter	3.23	1.20E-04	4.66	1.82E-08	NDE		2.75	1.54E-03	NDE	
Z0928	*galT*	Galactose-1-phosphate uridylyltransferase	3.24	7.98E-07	4.58	1.59E-12	NDE		2.92	1.32E-05	NDE	
Z3499	*glpA*	Glycerol 3-phosphate dehydrogenase (a subunit)	4.13	3.90E-06	2.50	1.07E-02	5.21	4.03E-09	3.42	2.20E-04	4.15	4.64E-06
Z3500	*glpB*	Glycerol 3-phosphate dehydrogenase (b subunit)	4.33	4.60E-06	NDE		5.01	1.05E-07	2.20	3.19E-02	3.77	1.06E-04
Z3501	*glpC*	Glycerol 3-phosphate dehydrogenase (c subunit)	3.91	2.15E-05	NDE		3.84	4.04E-05	NDE		2.51	1.03E-02
Z4786	*glpD*	Glycerol 3-phosphate dehydrogenase	3.62	8.99E-05	2.65	7.55E-03	4.98	4.13E-08	4.75	1.79E-07	4.23	4.61E-06
Z5472	*glpF*	Glycerol uptake facilitator protein	4.02	3.20E-09	3.66	1.38E-07	4.02	4.65E-09	5.33	2.65E-15	2.63	2.44E-04
Z5471	*glpK*	Glycerol kinase	3.80	3.61E-06	2.89	8.21E-04	3.87	3.11E-06	4.33	1.20E-07	2.38	6.39E-03
Z3498	*glpT*	Glycerol 3-phosphate transporter	5.65	1.72E-09	3.47	5.41E-04	5.44	8.86E-09	3.71	1.57E-04	4.05	3.23E-05
Z4805	*gntK*	Gluconokinase	NDE		2.44	3.04E-04	NDE		NDE		NDE	
Z5919	*gntP*	High-affinity gluconate transporter	5.85	5.74E-16	3.17	4.16E-05	NDE		NDE		NDE	
Z4770	*gntT*	Gnt-I system high-affinity gluconate transporter	NDE		2.52	1.95E-07	NDE		NDE		NDE	
Z4804	*gntU*	Gnt-I system low-affinity gluconate transporter	NDE		2.35	1.99E-03	NDE		NDE		NDE	
Z1076	*grxA*	Glutaredoxin 1	2.97	1.14E-06	5.31	2.61E-19	3.99	2.46E-11	4.51	3.83E-14	4.00	2.26E-11
Z4879	*hpR*	Phosphocarrier protein	NDE		NDE		2.56	5.73E-04	2.14	7.02E-03	2.32	1.63E-03
Z1389	*hyaA*	Hydrogenase I (small subunit)	2.62	3.08E-03	2.51	6.62E-03	6.24	3.71E-14	2.38	8.91E-03	5.66	9.23E-12
Z1390	*hyaB*	Hydrogenase I (large subunit)	2.58	3.41E-03	2.48	6.98E-03	6.32	1.14E-14	2.72	2.20E-03	5.69	5.17E-12
Z1391	*hyaC*	Hydrogenase I (B-type cytochrome subunit)	2.31	9.30E-03	2.39	9.57E-03	5.77	1.92E-12	2.45	6.16E-03	5.05	1.15E-09
Z1392	*hyaD*	Hydrogenase I (maturation protease)	2.22	1.16E-03	2.42	7.60E-03	5.47	1.45E-11	2.29	9.99E-03	4.81	4.38E-09
Z4350	*hybA*	Hydrogenase-2 (small subunit)	4.01	6.41E-09	2.92	5.37E-05	2.77	1.24E-04	NDE		2.27	2.01E-03
Z4349	*hybB*	Ni/Fe component of hydrogenase-2	4.24	1.21E-09	3.13	1.59E-05	2.86	8.54E-05	NDE		2.27	2.36E-03
Z4348	*hybC*	Hydrogenase-2 (large subunit)	3.83	3.42E-08	2.8	1.09E-04	2.65	2.71E-04	NDE		2.00	7.53E-03
Z4347	*hybD*	Hydrogenase maturation protease	3.39	1.11E-06	2.33	1.68E-03	2.22	2.74E-03	NDE		NDE	
Z5632	*malE*	Maltose/maltodextrin transport system (substrate-binding protein)	NDE		3.22	4.60E-05	NDE		2.41	3.70E-03	NDE	
Z5631	*malF*	Maltose/maltodextrin transport system (permease protein)	NDE		2.71	9.82E-06	NDE		NDE		NDE	
Z5630	*malG*	Maltose/maltodextrin transport system (permease protein)	NDE		2.79	3.81E-05	NDE		NDE		NDE	
Z5633	*malK*	Multiple sugar transport system (ATP-binding protein)	NDE		3.99	7.37E-08	NDE		2.36	3.38E-03	NDE	
Z2860	*manX*	PTS system mannose-specific IIAB component	NDE		2.69	4.64E-08	NDE		NDE		NDE	
Z2861	*manY*	PTS system mannose-specific IIC component	NDE		2.81	5.05E-09	NDE		NDE		NDE	
Z2862	*manZ*	PTS system mannose-specific IID component	NDE		2.66	1.49E-07	NDE		NDE		NDE	
Z3404	*mglA*	Methyl-galactoside transport system (ATP-binding protein)	2.98	5.30E-04	4.04	1.89E-06	2.84	1.28E-03	3.38	8.10E-05	NDE	
Z3405	*mglB*	Methyl-galactoside transport system (substrate-binding protein)	3.75	1.06E-06	4.77	4.85E-10	2.76	6.21E-04	4.09	1.00E-07	NDE	
Z3403	*mglC*	Methyl-galactoside transport system (permease protein)	3.02	4.18E-05	3.33	7.49E-06	3.06	4.16E-05	2.85	1.52E-04	NDE	
Z0824	*nagA*	N-acetylglucosamine 6-phosphate deacetylase	2.37	7.39E-06	2.95	2.19E-08	NDE		NDE		NDE	
Z0825	*nagB*	Glucosamine 6-phosphate deaminase	3.44	1.74E-07	4.49	7.20E-12	NDE		NDE		NDE	
Z0826	*nagE*	PTS system N-acetylglucosamine-specific IIABC component	3.31	4.52E-08	4.11	9.49E-12	NDE		2.25	3.91E-04	NDE	
Z4583	*nanA*	N-acetylneuraminate lyase	7.79	1.62E-18	5.14	2.72E-08	3.55	2.07E-04	5.90	8.61E-11	NDE	
Z4582	*nanT*	Sialic acid transporter	6.32	1.03E-13	3.85	1.80E-05	2.30	1.71E-02	5.18	2.28E-09	NDE	
Z3463	*napA*	Nitrate reductase (catalytic subunit)	2.78	2.25E-07	NDE		2.66	1.03E-06	NDE		2.77	3.14E-07
Z3460	*napB*	Cytochrome v-type protein NapB	NDE		NDE		2.35	4.67E-03	2.24	7.15E-03	NDE	
Z3461	*napH*	Ferredoxin-type protein NapH	NDE		NDE		2.33	3.08E-03	NDE		2.15	6.29E-03
Z2001	*narG*	Nitrate reductase (α subunit)	NDE		NDE		4.50	1.93E-23	NDE		4.97	1.46E-28
Z2002	*narH*	Nitrate reductase (β subunit)	NDE		NDE		2.97	2.34E-15	NDE		3.36	1.58E-19
Z2004	*narI*	Nitrate reductase (γ subunit)	NDE		NDE		2.95	8.43E-11	NDE		3.30	2.02E-13
Z2003	*narJ*	Nitrate reductase molybdenum cofactor	NDE		NDE		3.23	3.23E-11	NDE		3.67	2.51E-14
Z4726	*nirB*	Nitrite reductase (large subunit)	NDE		NDE		NDE		NDE		2.06	9.01E-07
Z5671	*nrfC*	Nitrite reductase (formate-dependent)	NDE		NDE		5.35	1.49E-13	2.63	8.52E-04	5.20	7.59E-13
Z5673	*nrfE*	Cytochrome c-type biogenesis protein NrfE	NDE		NDE		3.43	2.08E-06	NDE		3.35	3.59E-06
Z5674	*nrfF*	Formate-dependent nitrite reductase complex subunit NrfF	NDE		NDE		3.02	1.58E-04	NDE		3.06	1.23E-04
Z5675	*nrfG*	Formate-dependent nitrite reductase complex subunit NrfG	NDE		NDE		2.68	4.04E-05	NDE		2.78	1.73E-05
Z3541	*nuoH*	NADH-quinone oxidoreductase subunit H	NDE		NDE		NDE		2.45	5.24E-04	NDE	
Z3540	*nuoI*	NADH-quinone oxidoreductase subunit I	NDE		NDE		2.04	5.31E-03	2.72	9.55E-05	NDE	
Z3539	*nuoJ*	NADH-quinone oxidoreductase subunit J	NDE		NDE		2.02	5.51E-03	2.82	1.86E-05	NDE	
Z3538	*nuoK*	NADH-quinone oxidoreductase subunit K	NDE		NDE		NDE		2.89	4.64E-06	NDE	
Z3537	*nuoL*	NADH-quinone oxidoreductase subunit L	NDE		NDE		NDE		2.76	1.98E-05	NDE	
Z3536	*nuoM*	NADH-quinone oxidoreductase subunit M	NDE		NDE		NDE		3.04	1.26E-06	NDE	
Z4758	*pckA*	Phosphoenolpyruvate carboxykinase	3.54	2.78E-10	2.60	8.68E-06	2.86	6.67E-07	3.44	1.30E-09	NDE	
Z3401	*preT*	Dihydropyrimidine dehydrogenase	2.94	2.12E-06	2.48	1.19E-04	6.46	4.01E-28	2.32	3.81E-04	5.40	9.99E-20
Z3980	*proW*	Glycine betaine/proline transport system	2.36	8.30E-03	NDE		NDE		-2.58	4.47E-03	NDE	
Z3981	*proX*	Glycine betaine/proline transport system	2.74	6.12E-04	NDE		NDE		-2.1	1.24E-02	NDE	
Z3653	*ptsN*	PTS system fructose-specific IIB-like component	NDE		NDE		NDE		NDE		2.55	4.69E-03
Z5250	*rbsA*	Ribose transport system ATP-binding protein	6.24	4.46E-23	5.83	7.52E-20	2.84	2.63E-05	5.80	9.53E-20	4.60	9.20E-13
Z5252	*rbsB*	Ribose transport system substrate-binding protein	4.23	1.81E-08	2.16	9.34E-03	NDE		2.59	1.10E-03	NDE	
Z5251	*rbsC*	Ribose transport system permease protein	6.49	5.50E-29	5.18	2.95E-18	4.04	1.99E-11	6.28	1.33E-26	5.00	3.27E-17
Z5249	*rbsD*	D-ribose pyranase	6.17	2.40E-23	5.28	5.82E-17	3.40	1.91E-07	6.10	2.29E-22	4.81	3.25E-14
Z5253	*rbsK*	Ribokinase	3.82	6.03E-12	2.13	3.67E-04	NDE		2.81	9.77E-07	NDE	
Z2857	*sdaA*	L-serine dehydratase	2.18	2.59E-15	NDE		2.46	3.92E-19	2.47	4.52E-19	2.25	3.44E-16
Z4114	*sdaB*	L-serine dehydratase (deaminase)	4.36	2.42E-18	2.93	1.87E-08	NDE		NDE		NDE	
Z4113	*sdaC*	Serine transporter	4.59	1.85E-30	3.72	8.52E-20	NDE		2.39	1.43E-08	NDE	
Z0877	*sdhA*	Succinate dehydrogenase (flavoprotein subunit)	NDE		NDE		NDE		2.12	5.16E-03	NDE	
Z0878	*sdhB*	Succinate dehydrogenase (iron-sulfur subunit)	NDE		NDE		NDE		2.10	1.39E-02	NDE	
Z0876	*sdhD*	Succinate dehydrogenase (membrane anchor subunit)	NDE		NDE		NDE		2.04	2.05E-03	NDE	
Z0880	*sucA*	2-oxoglutarate dehydrogenase E1 component	NDE		NDE		NDE		2.60	6.80E-04	NDE	
Z0881	*sucB*	2-oxoglutarate dehydrogenase E2 component	NDE		NDE		NDE		2.91	3.11E-04	NDE	
Z0882	*sucC*	Succinyl-CoA synthetase (β subunit)	NDE		NDE		NDE		2.93	2.18E-04	NDE	
Z0883	*sucD*	Succinyl-CoA synthetase (α subunit)	NDE		NDE		NDE		2.89	1.78E-04	NDE	
Z5501	*talC*	Fructose 6-phosphate aldolase 2	2.45	3.24E-05	NDE		2.61	1.21E-05	NDE		2.16	3.41E-04
Z4469	*tdcB*	Threonine dehydratase	6.04	1.04E-09	NDE		6.43	7.98E-11	NDE		4.56	8.29E-06
Z4468	*tdcC*	Threonine/serine transporter	4.99	3.98E-07	NDE		5.02	3.93E-07	NDE		3.14	2.54E-03
Z4467	*tdcD*	Propionate kinase	5.22	2.18E-07	NDE		5.89	4.20E-09	2.29	3.87E-02	4.22	4.89E-05
Z4466	*tdcE*	Formate C-acetyltransferase	4.54	3.69E-05	NDE		5.05	4.69E-06	2.43	4.10E-02	3.79	8.59E-04
Z5203	*tnaA*	Tryptophanase	3.16	6.15E-03	NDE		4.73	2.43E-05	2.48	3.88E-02	3.62	1.69E-03
Z5204	*tnaB*	Low affinity tryptophan permease	NDE		NDE		3.28	2.20E-05	NDE		NDE	
Z4444	*uxaA*	Altronate hydrolase	5.41	1.05E-13	3.97	1.52E-07	NDE		NDE		NDE	
Z2184	*uxaB*	Tagaturonate reductase	4.08	9.81E-13	2.19	3.95E-04	NDE		NDE		NDE	
Z4445	*uxaC*	Glucuronate isomerase	6.19	2.40E-19	4.89	4.88E-12	NDE		NDE		NDE	
Z5920	*uxuA*	Mannonate hydrolase	7.50	3.08E-25	5.27	1.83E-12	2.86	2.82E-04	2.46	1.98E-03	NDE	
Z5921	*uxuB*	Mannonate oxidoreductase	6.68	2.40E-22	4.21	4.35E-09	2.41	1.52E-03	NDE		NDE	
Z4990	*xylA*	Xylose isomerase	NDE		3.45	4.67E-07	3.22	2.57E-06	4.60	3.99E-12	2.04	4.53E-03
Z4989	*xylB*	Xylulokinase	NDE		2.02	5.33E-03	NDE		2.85	2.43E-05	NDE	
Z4991	*xylF*	D-xylose transport system (substrate-binding protein)	NDE		4.83	3.40E-09	3.48	2.93E-05	5.77	3.03E-13	2.54	3.06E-03
Z4992	*xylG*	D-xylose transport system (ATP-binding protein)	NDE		NDE		NDE		2.08	4.14E-03	NDE	
Z4993	*xylH*	D-xylose transport system (permease protein)	NDE		NDE		2.03	1.36E-03	2.07	1.01E-03	NDE	
Z5717	*yjdE*	Arginine:agmatine antiporter	2.43	8.29E-03	NDE		3.76	1.94E-05	NDE		3.19	3.64E-04
Z0461	*z0461*	Fucose transporter (optimal import)	2.66	1.23E-03	3.00	3.12E-04	2.33	6.92E-03	4.75	1.65E-09	NDE	3.61E-04
Z1245	*z1245*	Putative transporter	NDE		NDE		2.02	1.93E-03	NDE		2.93	9.54E-05
Z2575	*z2575*	Tat-targeted selenite reductase subunit YnfE	2.86	2.35E-05	NDE		2.97	1.35E-05	NDE		2.68	2.24E-04
Z2576	*z2576*	Tat-targeted selenite reductase subunit YnfF	2.55	1.58E-04	NDE		3.31	7.19E-07	NDE	1.65E-09	2.57	3.61E-04
Z2577	*z2577*	Tat-targeted selenite reductase subunit YnfG	2.75	2.04E-04	2.04	1.04E-02	3.44	3.00E-06	NDE		2.78	5.41E-04
Z3394	*z3394*	MFS transporter	NDE		2.75	9.22E-03	2.42	2.39E-02	4.54		3.92	4.73E-03
Z3715	*z3715*	Ethanolamine utilization cobalamin adenosyltransferase	NDE		NDE		3.94	1.25E-03	NDE	6.32E-05	4.28	4.33E-11
Z4207	*z4207*	Xanthine dehydrogenase iron-sulfur-binding subunit	NDE		NDE		2.86	2.14E-06	NDE		2.15	1.85E-04
Z4464	*z4464*	L-serine dehydratase	4.15	1.51E-04	NDE		4.25	1.30E-04	NDE		3.22	1.87E-06
Z4875	*z4875*	PTS system galactitol-specific IIA component	2.44	8.99E-06	NDE		3.84	2.25E-12	2.36	1.45E-05	3.61	3.91E-09
Z4876	*z4876*	PTS system galactitol-specific IIB component	2.98	2.87E-07	NDE		3.79	6.23E-11	2.68		3.43	
Z4877	*z4877*	PTS system galactitol-specific IIC component	2.22	2.94E-05	NDE		2.37	6.12E-06	NDE	3.13E-06	NDE	7.53E-05

*NDE* not differentially expressed

The transcription of genes involved in the transport and assimilation of amino acids also appeared to be commonly induced in EHEC EDL933 in the three DCs (Fig. 4, Additional file 3: Table S7). The genes encoding i) the aerobic transport of aspartate and deamination of aspartate to fumarate (*dctA* and *aspA*, respectively), ii) the conversion of serine (*sdaA*) and tryptophane (*tnaA*) to pyruvate, iii) the degradation of threonine and serine *via* the propanoate pathway (*tdcE* and *tdcD*, respectively) and iv) the hydrolysis of asparagine to aspartate (*asnA*) were found commonly over-expressed in EHEC EDL933 (Table 3). Note that DctA is also the aerobic transporter of common C_4_-dicarboxylates (succinate, fumarate and malate). All these genes were more transcribed in EDL933 incubated in rumen and small intestine contents (log2FC > 3) than in rectum content, except *dctA* and *sdaA* (Table 3). In addition, *glpF* and *glpT* (transport of glycerol and glycerol 3-phosphate [G3P], respectively), and *glpK* (ATP-dependent glycerol kinase) were commonly induced in EDL933 incubated in the three DCs (Table 3).

#### Carbon sources common to the posterior intestinal contents (small intestine and rectum contents)

The rumen is not considered as a site of EHEC colonization while the small intestine and the rectum constitute more favorable environments for EHEC multiplication. Therefore, we focused here on the genes commonly up-regulated in the two posterior DCs. Genes included in “Carbohydrate transport and metabolism”, “Amino acid transport and metabolism” and “Energy production and conversion” COG categories were found commonly up-regulated in EHEC at the mid- (81 genes) and late-exponential (79 genes) growth phases.

Up-regulation of the genes encoding proteins involved in the transport or metabolism of N-acetylneuraminic acid (Neu5Ac, *nanAT*), N-acetylglucosamine (GlcNAc, *nagABE*) and galactose (*galKMPT* and *mglABC*) was unique to EDL933 incubated in posterior DCs (Table 3). The genes *fucAIOP* and *z0461* encoding proteins involved in the transport and assimilation of fucose were also over-expressed in both small intestine and rectum contents during the two growth phases, while only *fucA* and *fucO* appeared over-expressed in rumen content (Table 3). In addition to these genes associated with mucus-derived carbohydrate assimilation, genes encoding proteins involved in the transport and/or utilization of arabinose (*araABDFG*), fructose (*fruABK*), gluconate (*gntKPTU*) and hexuronate (galacturonate and glucuronate) (*uxaABC* and *uxuAB*) also exhibited increased expression in the posterior compartments. Noteworthy, i) *uxaABC* and *gntKPTU* were exclusively expressed during the mid-exponential growth phase and ii) the *fru* genes were more induced during incubation in small intestine than in rectum content. In addition to carbohydrates, EHEC EDL933 also induced pathways involved in the transport (*sdaC*) and catabolism (*sdaB*) of serine (Table 3).

#### Specific carbon sources in rectum contents

Because the rectum is the major site of EHEC colonization in the bovine GIT, particular attention was paid to the genes specifically up-regulated in EHEC during incubation in rectum content. The transcription of the genes *malEFGK* and *manXYZ* encoding maltose and mannose uptake, respectively, was exclusively induced in EDL933 during incubation in rectum content, mainly during the mid-exponential growth phase (Table 3). The genes encoding proteins required for the transport and assimilation of gluconate were all up-regulated in rectum content at mid-exponential growth phase (Table 3). In addition, the transcription of the genes encoding the transport (*xylF*) and catabolism (*xylAB)* of xylose was induced in EHEC EDL933 during the mid-exponential growth phase in rectum but not in small intestine contents (Table 3). Note also that i) all the *aga* genes were up-regulated in both mid- and late-exponential growth phases in rectum content but only during the late-exponential growth phase in small intestine content, ii) the fold change increase in *aga* genes expression during the late-exponential growth phase was higher in rectum than in small intestine or rumen contents, and iii) the genes *xylF*, *xylA*, *fucA, fucO* and *rbsACD* (transport or assimilation of xylose, fucose or ribose) had higher transcripts levels in EDL933 incubated in rectum content than in the two other DCs during the late-exponential growth phase (Table 3). The genes coding for the transport and assimilation of galactose were expressed at a higher level in rectum than in small intestine contents (except *mglC* during the late-exponential growth phase). Importantly, the genes encoding the fucose-sensing two component system *fusK* and *fusR* (*z0462* and *z0463*) were over-expressed in EHEC grown in rectum content during both growth phases while only the regulator *fusR* was up-regulated in EHEC incubated in small intestine content during the mid-exponential growth phase.

To broaden this analysis, the expression of the genes mentioned above was also directly compared in bacteria grown in rectum vs small intestine content and rectum vs rumen content (Additional file 3: Table S8). The results confirmed the specific up-regulation of several genes involved in the transport or assimilation of GalNac, fucose, maltose, ribose and xylose (Additional file 3: Table S8). However, this analysis did not allow us to confirm the specific up-regulation of mannose and gluconate transport and/or assimilation encoding genes in the rectum content.

Taken together, these results suggest an important role for xylose, fucose, GalNac, ribose and maltose in the colonization of the cattle rectum by EHEC.

### Genes up-regulated relative to transport and assimilation of ethanolamine

Ethanolamine (EA) is an important nitrogen source that confers a growth advantage to EHEC EDL933 in bovine small intestine content [20]. Here, we focused on the genes encoding EA transport and assimilation in the different DCs. As expected, the transcription of the *eut* genes was induced in small intestine content during the late-exponential growth phase (Table 3). Interestingly, most of the *eut* genes also exhibited an increased expression in EHEC incubated in rumen content at 6h of incubation whereas only 7 of the 14 *eut* genes were up-regulated in EHEC incubated in rectum content (Table 3). Note that *eutH,* encoding an active transport of EA across the bacterial membrane, was up-regulated in both small intestine and rumen contents but not in rectum content. None of the *eut* genes were found differentially expressed in EHEC during the mid-exponential growth phase in small intestine and rectum contents.

### Genes up-regulated relative to C_4_-dicarboxylate transporters

The transport of common C_4_-dicarboxylates (fumarate, malate, succinate) as well as aspartate under aerobic and anaerobic conditions is well documented [22]. In this study, the gene *dcuB* (anaerobic antiporting of succinate against fumarate) was up-regulated in EHEC incubated in the three DC whereas the transcription of *dcuA* (anaerobic antiporting of succinate against aspartate) was only induced during incubation in small intestine and rectum contents (Table 3). Surprisingly, *dctA* (aerobic uptake of C_4_-dicarboxylates) was up-regulated in EHEC during incubation in all the DCs tested, including rumen and rectum contents under anaerobiosis, suggesting differences in *dctA* regulation depending on the bacterial growth medium. Interestingly, succinate was excreted by EHEC EDL933 in small intestine content (1.1 mM ± 0.13 and 5.3 mM ± 0.13 succinate at 3 and 6h of incubation, respectively), but not in rumen or rectum contents, suggesting that antiporting of aspartate against succinate only occurred in the bovine small intestine.

### Genes relative to energy production and conversion

An in-depth analysis of the genes associated with respiratory pathways activated by EHEC EDL933 in the three DCs was undertaken. As shown in Table 3, common and distinct respiratory systems were induced by the bacteria according to the bovine DCs. The genes *fdnG* and *fdoHI* (formate dehydrogenase-N and -I respectively), *frdABCD* (fumarate reductase), *glpABD* (G3P dehydrogenase), hyaABCD and hybABC (hydrogenase-1 and -2 respectively) were commonly up-regulated in EHEC EDL933 in the three DCs. Otherwise, *appBC* (cytochrome bd oxydase), *dmsABC* (DMSO reductase), *napAH* and *narGHIJ* (nitrate reductases) as well as *nrfEFG* (formate-dependent nitrite reductase) were up-regulated in both rumen and small intestine contents (Table 3). Furthermore, the gene *cydB* (cytochrome bd oxidase) was up-regulated in both small intestine and rectum contents. The transcription of the genes *sdhABC* (succinate dehydrogenase) and *nuoHIJKLM* (NADH oxidoreductase) was only activated in rectum content except *nuoI* and *nuoJ* which were also up-regulated in small intestine content. Note i) the redundancy of formate dehydrogenases (*fdn* and *fdo*), hydrogenases (*hya* and *hyb*) and nitrate reductases (*nap* and *nar*) encoding genes and ii) that, surprisingly, genes encoding proteins involved in aerobic respiration were found up-regulated in EHEC EDL933 incubated in rumen (*fdoHI*, *appBC* and *glpD*) and rectum contents (*cydB*, *fdoHI*, *sdhABC, nuoHIJKLM* and *glpD*) under anaerobic culture conditions.

## Discussion

Mucin, the main constituent of the mammalian intestinal mucus, is a filamentous glycoprotein rich in oligosaccharides (up to 80% of the mucin biomass) and represents a niche for bacterial colonization of the intestine [23, 24]. Indeed, mucus is constantly released into the mammal GIT and degraded by a subset of commensal bacteria able to breakdown mucin into sugars. Monomers of the six main carbohydrates present in the mucus covering the bovine small intestine epithelium (galactose, GlcNAc, GalNAc, fucose, mannose and Neu5Ac) [25] have been previously detected in bovine small intestinal contents [17]. Importantly, galactose, GlcNAc, mannose and Neu5Ac conferred a competitive growth advantage to EHEC in bovine small intestine content [17]. However, the carbohydrate composition of the mucus layer covering other bovine digestive compartments has not yet been documented. Transcriptome analyses suggested that EHEC EDL933 could assimilate galactose, GlcNAc and Neu5Ac in posterior bovine DCs (small intestine and rectum contents) whereas fucose and GalNac could be used by the bacteria all along the bovine GIT. In addition, our results suggest that fucose and GalNac would be more important substrates for EHEC in the rectum. Altogether, these data suggest differences in mucus-derived carbohydrate composition and availability depending on bovine digestive compartments. Accordingly, renewal rate, carbohydrate composition and thickness of the mucus layer vary for different mammal gut regions. For example, nitrogen and organic matter content of bovine mucus is known to be different between the ileum and colon [26].

Survival of *E. coli* in the stationary growth phase depends, at least partially, on nutrients that are still available after the bacterial exponential growth phase. In this study, comparison of gene expression between mid- and late-exponential growth phases allows us to propose a sequential utilization of mucus-derived carbohydrates by EHEC in the small intestine content: galactose and GlcNAc would be rapidly assimilated by EHEC and exhausted when the bacteria reached the late-exponential growth phase; fucose and Neu5Ac would be used by EHEC up to the late-exponential growth phase whereas GalNAc would be assimilated in a second step when galactose and GlcNAc are exhausted. Accordingly, it has been shown that *E. coli* MG1655 also displays a preference for specific mucus derived monosaccharides at different stages of mouse intestine colonization: genes involved in GlcNAc catabolism were essential for colonization initiation whereas genes involved in fucose catabolism were required for maintenance [27]. Taken together, our results strongly suggested that mucus-derived carbohydrates i) are important carbon sources for successful survival of EHEC in the bovine gut and ii) would be assimilated by EHEC in order of preference for sugars.

Additional carbohydrates can be assimilated by EHEC in the mammal GIT. For example, arabinose, hexuronate and ribose are known to be used by EHEC EDL933 to colonize streptomycin-treated mice [28]. The concentration of free mono- and di-saccharides released from the degradation of the animal diet is generally low in the rumen of cattle because the endogenous microbiota is very efficient in assimilating these substrates [29, 30]. However, in addition to mucus-derived carbohydrates, these sugars may originate from other host derived glycans such as epithelial glycocalyx present underneath the mucus layer or from prokaryotic and eukaryotic lysed cell components. They may also be released from the degradation of dietary polysaccharides that have escaped ruminal hydrolysis. In this study, we hypothesized that EHEC EDL933 assimilated xylose, ribose, mannitol and galactitol all along the bovine GIT whereas arabinose, fructose, gluconate and hexuronates would be exclusively used by the bacteria in small intestine and rectum contents. Ribose is one of the preferred sugars used by *E. coli* and the catabolism of xylose and ribose allows the synthesis of precursors constituting important substrates for the pentose phosphate pathway [27, 28]. Interestingly, mutation in the ribose assimilation pathway causes a defect in the ability to colonize for EHEC, but not for commensal *E. coli* in the mouse intestine [28]. Gluconate is also metabolized by *E. coli* in the mice intestine and its distribution in drinking water impacts mice colonization by EHEC EDL933 [31, 32]. Noteworthy, gluconate transport and utilization were also induced by the probiotic bacterium *Propionibacterium freudenreichii* in the colonic environment *in vivo* compared with artificial medium *in vitro* [33]. Gluconic acid is present in plant products, but also in mammal intestinal mucus [27, 33]. Therefore, it is reasonable to speculate that gluconate could be present in the bovine small intestine and rectum and potentially assimilated by EHEC at these sites.

Gene transcription analysis during incubation in the bovine rectum provided valuable information since this intestinal section is referred to as the major site of O157:H7 EHEC colonization in adult cattle [19]. As mentioned above, EHEC activated the transcription of genes required for the assimilation of galactose, GlcNAc, Neu5Ac, fucose and GalNac in rectum contents. Importantly, we showed that the genes encoding PTS for uptake or assimilation of maltose were exclusively over-expressed in EHEC EDL933 during incubation in rectum content, suggesting a specific role for this sugar at this site. Maltose also plays an important role in the colonization of mice by EHEC and the utilization of this sugar confers a competitive advantage to the pathogen during the initiation of colonization when competing with commensal *E. coli* [31]. Maltose can have a dietary origin and be generated through the degradation of starch present in the bovine ration by the endogenous microbiota. However, in the bovine rectum, maltose would be provided by lysed cells releasing their endogenous glycogen. Indeed, glycogen storage is widespread in gut microbiota because it represents a useful strategy for rapid growth in a very competitive ecosystem [31, 34]. Also, our study strongly suggested an important role for xylose, GalNac and fucose in EHEC colonization of the rectum. Fucose was previously described as a critical carbon source for the maintenance of EHEC in the rectum of adult cattle [19]. Accordingly, our results showed that the transcription of most *fuc* genes, including *fucP* (fucose/H^+^ symporter) and *z0461* (optimal fucose import), were activated in EHEC EDL933 incubated in rectum content. Note that i) a *z0461* deletion reduces the expression of *fuc* genes and decreases the capacity of EHEC to use fucose to grow [35], and ii) *fusK* and *fusR,* encoding a fucose sensing two-component system, were up-regulated in EHEC during incubation in rectum content. The FusKR system is known to repress the expression of *z0461* and the *fuc* genes during incubation of EHEC in minimal media supplemented with fucose as a sole carbon source [35]. The up-regulation of *fuc*, *z0461* and *fus* genes in rectum content, a much more complex medium, strongly suggested differences in FusKR regulation depending on compounds or chemical conditions in bacterial environments. Further studies are needed to analyze the regulation of *fuc* gene expression under our experimental conditions.

The gluconeogenesis pathway contributes to bacterial fitness *in vivo* and utilization of gluconeogenic substrates is required for a maximal colonization of the mouse intestine by EHEC and for maintaining EHEC in bovine small intestine content [21, 36]. Previous studies from our laboratory showed that i) EHEC EDL933 uses preferentially mucus-derived sugars during the exponential growth phase in bovine small intestine content and then assimilates gluconeogenic substrates when sugars were exhausted, and ii) gluconeogenic substrates such as aspartate, serine and glycerol are present in small intestine content [17, 21]. The present work showed that amino acids (aspartate and serine), glycerol and G3P would also probably be used all along the bovine GIT as genes encoding their transport and/or metabolism were activated in all the DCs tested. It is well documented that bovine mucin contains a high concentration of glutamic and aspartic acids, threonine and serine [25]. Furthermore, serine and glycerol are part of phospholipids constituting eukaryotic and prokaryotic cell membranes and thus could be released into bovine gut contents during cell renewal.

Ethanolamine (EA) is included in phosphatidylethanolamine, the most abundant phospholipid in eukaryotic and prokaryotic cell membranes. Free EA, used as a nitrogen source, confers a nutritional advantage to EHEC and contributes to the survival of the pathogen in bovine small intestine content [20]. In a previous study, we showed that the concentration of free EA is much more important in bovine small intestine (2.2 mM) than in rumen (7.7 nM) or colon (17.3 nM) contents [20]. In the present report, most of the *eut* genes were found up-regulated in EHEC incubated in small intestine and rumen contents but only half of them in rectum content. However, the weak concentration of EA in rumen content strongly suggested that EA probably cannot promote EHEC growth in the rumen of cattle. Kendall *et al.* [37] showed that EA could be a signal for gene regulation, and could then play complex roles in metabolism, cell-to-cell signaling and bacterial virulence. However, the specific mechanisms of gene regulation by EA remain to be completely elucidated. The results of our study suggest that EA could positively regulate the expression of genes whose products are involved in EHEC survival in the rumen, but further studies will be necessary to elucidate its role in the cattle rumen.

The mammalian intestine is not strictly anaerobic but contains distinct microaerobic and anaerobic regions due to i) dynamic cycles of oxygen diffusion from intestinal epithelium and ii) oxygen consumption by facultative anaerobes [38–41]. It is well documented that nitrate is the preferred terminal electron acceptor during nitrate respiration in *E. coli* when oxygen became limited in the mammal intestine [22, 40, 42]. In contrast, fumarate constitutes the terminal electron acceptor during fumarate respiration, when oxygen is absent and nitrate is present at low levels [22, 40, 42]. In this study, EHEC EDL933 up-regulated simultaneously several aerobic and anaerobic respiratory systems in all the DCs tested, suggesting that EHEC colonization of the bovine GIT requires respiratory flexibility. Accordingly, microaerobic (oxygen) and anaerobic (nitrate and fumarate) niches are equally crucial for successful EHEC colonization of the mouse intestine [40, 41]. Taken together, our transcriptome analyses strongly suggested that EHEC could potentially use distinct dehydrogenases and reductases during its transit in the bovine GIT. This could be due to availability and fluctuation of electron donors (H_2_, formate, succinate, G3P) and acceptors (oxygen, nitrate, fumarate) in the animal gut. The redundancy and complexity in the respiratory systems activated by EHEC could facilitate a rapid adaptation of the bacteria in changing intestinal environments.

Fumarate, detected in the mammalian intestine at low levels, is endogenously generated by bacteria via central metabolism. Interestingly, the transcription of the genes encoding anaerobic antiporting of succinate against aspartate or fumarate described by Unden *et al.* [22] (*aspA, dcuA, dcuB*) was activated in EHEC incubated in the three DCs. We have previously shown that EHEC assimilate aspartate present in bovine small intestinal content [21] and we demonstrated in this study that EHEC EDL933 excreted succinate in small intestine content (but not in rumen or rectum contents) whereas fumarate was undetectable before and after EHEC incubation (results not shown). Taken together, these results strongly suggested i) the anaerobic antiporting of external aspartate against succinate due to the DcuA antiporter and ii) a supply of intracellular fumarate due to aspartate deamination under anaerobiosis. We speculated that the supply of intracellular fumarate via aspartate deamination might represent an additional source of electron acceptors used by EHEC during anaerobic fumarate respiration in the bovine small intestine. This hypothesis was recently confirmed during incubation of EHEC in small intestine content [43].

## Conclusions

In conclusion, EHEC EDL933 showed a high flexibility in activation of genes involved in respiratory pathways and assimilation of carbon and nitrogen sources, most of them from animal origin. Such a flexibility may allow the bacterium to adapt and survive in the various segments of the bovine GIT with different nutrient composition. Our results open new avenues to find strategies designed to specifically reduce intestinal nutrients required for *E. coli* O157:H7 colonization. To this end, administration of probiotics with a high efficiency in the assimilation of the nutrients preferred by EHEC to ruminants may be an effective pre-harvest strategy to limit EHEC carriage and shedding by cattle.

## Methods

### Bacterial strains characteristics

The wild type *E. coli* strains and the rifampicin-resistant (Rif^R^) mutants used in this study were described in Additional file 1: Table S1. Bacteria were routinely cultured on Luria Bertani (LB) agar plates. Spontaneous Rif^R^ mutants of *E. coli* NV95 and Sakai strains were isolated by culturing the wild-type strain on LB agar plates containing 100 μg mL^-1^ of rifampicin. The wild-type strains and their corresponding spontaneous Rif^R^ mutants showed identical growth curves when incubated in LB broth at 37°C with aeration (results not shown).

### Origin and sampling of digestive contents

Eight healthy young bulls (*Bos taurus*) from the “Herbipole experimental INRA farm”, approximately 2 years of age and 550 kg mean weight, were slaughtered in the experimental slaughterhouse of the “Herbipole” (National Institute for Agronomic Research [INRA], Saint-Genès-Champanelle, France) (Slaughterhouse Permit number: 63345001). The bulls were raised according to current INRA ethical guidelines for animal welfare and the experiments were approved by the local ethics committee (Permit Number: C6334517). Digestive contents (DCs) were collected from each bull fed a mixed diet containing hay (80%) and concentrate (20%) (major compounds: rapeseed [26%], maize [20%], barley [14%], sunflower [8.5%], triticale colza [8.5%]). All DCs from rumen, small intestine, caecum and colon compartments were collected from each animal at slaughter while rectum contents were collected two days before slaughter by rectal palpation. Small intestine contents were collected from the jejunum and ileum removed in a single piece as previously described [20]. All DCs were rapidly collected and immediately brought to the laboratory. Small intestine contents were directly distributed in sterile tubes without any particular attention paid regarding anaerobiosis. The other contents were processed under strictly anaerobic conditions as previously described [44]. Briefly, rumen contents were filtered through four layers of cheesecloth to remove large feed particles. Caecum, colon and rectum contents were diluted 1:1 in reduced potassium phosphate buffer (50 mM potassium phosphate, resazurin 0.1%, 40 mM Na_2_CO_3_, 3 mM cysteine, pH 7.6) which was prepared in order to maintain a low redox potential [44]. Rumen, caecum, colon and rectum samples were distributed in sterile O_2_-free CO_2_-saturated Hungate tubes (Bellco). All these DC samples, containing the live endogenous microbiota and noted as unfiltered samples, were frozen at -80°C until use. These conditions were previously described as an appropriate way to maintain the viability of the bovine intestinal microbiota [7]. DC samples from the eight animals were pooled before use. Endogenous microbiota rifampicin sensibility was confirmed by spotting each pooled DC (100 μL) on Sorbitol Mac Conkey (SMAC) agar plates supplemented with 100 μg mL^-1^ rifampicin before incubation at 37°C for 24h.

To remove endogenous microbiota, the frozen unfiltered DC samples were thawed, centrifuged twice for 15 min at 10,000 × g, and supernatants were filtered through a Steritop and Stericup system with a membrane pore size 0.22 μm (Millipore). Filtrates, except small intestine filtrates, were placed in Hungate tubes which were left without stoppers in an anaerobic chamber (JACOMEX, Lyon, France) under 80-90 ppm of oxygen during three days at room temperature. This allowed the filtrates to be under anaerobic conditions. Tubes were then recapped before being removed from the anaerobic chamber, refiltered through 0.22 μm pore-size filters (Millipore) and placed into new O_2_-free CO_2_-saturated sterile Hungate tubes. The filtration efficacy was checked by inoculating an aliquot of the filtrates into LB agar plates before overnight incubation at 37°C. Filtered DCs were stored at room temperature until use.

### Inoculation of *E. coli* strains in unfiltered and filtered digestive contents

The wild type strains EDL933, Sakai, NV95 and BG1, and their respective spontaneous Rif^R^ mutants were inoculated from a single colony and incubated in LB medium without antibiotic or supplemented with rifampicin (100 μg mL^-1^), respectively, for 7h at 37°C with aeration. The cultures were then 50-fold diluted in filtered DCs and grown overnight at 39°C without aeration. The day after, the bacterial concentration was spectrophotometrically adjusted at 600 nm to ≈ 10^4^ bacteria mL^-1^ before inoculating unfiltered and filtered DCs. DCs were finally incubated at 39°C (internal bovine temperature) and i) under strict anaerobiosis with gentle shaking (rumen content) or without shaking (caecum, colon and rectum contents) or ii) under oxygen-limited conditions without shaking in small intestine content as previously described [21]. These conditions (temperature, shaking and oxygenation) were chosen to reflect the *in vivo* conditions for each bovine digestive compartment [7, 20, 21, 44]. At each time point (every two hours), an aliquot of unfiltered and filtered DC was 10-fold serially diluted in sterile phosphate buffered saline buffer (pH 7.4) and spotted on SMAC agar plates supplemented with rifampicin (100 μg mL^-1^) or without antibiotic, respectively. The plates were incubated overnight at 37°C before counting colony forming unit (CFU). Each experiment was replicated at least three times.

### pH and metabolite quantification

Metabolite concentration was quantified in unfiltered and filtered DCs. The samples were first centrifuged (10,000 × g for 10 min). For short chain fatty acid (SCFA) quantification in unfiltered DCs, supernatants were filtered (0.22 μm) and 30 μL of orthophosphoric acid (75%) were added to 1 mL of supernatant. Total SCFA concentrations were determined by gas chromatography. For succinate quantification in filtered DCs, 200 μL of ZnSO_4_ 5% and Ba (OH)_2_ 3 M were added to 800 μL of supernatant before quantification determined by high-performance liquid chromatography. All the analysis were performed by AFYREN INVESTMENT (Biopole Clermont Limagne, Saint Beauzire, France). pH measurements were performed for unfiltered DCs using a HI-8424N pH meter (HANNA instruments).

### RNA extraction and mRNA enrichment

The M9 minimal medium [45] was supplemented with glucose (40 mM), MgSO_4_ (1 mM), CaCl_2_ (0.1 mM) and trace metals (M9-Glc), and adjusted to ≈ pH 7.4. EHEC EDL933 inoculated from a single colony was incubated in filtered DCs under growth conditions described above, except that the initial bacterial concentrations were adjusted to ≈ 10^8^ cells mL^-1^ before inoculating rumen content, ≈ 10^6^ cells mL^-1^ before inoculating small intestine and rectum contents and ≈ 10^7^ cells mL^-1^ before inoculating M9-Glc (Additional file 1: Figure S1). The M9-Glc bacterial cultures were grown under oxygen-limited conditions. Three biological replicates were performed for each culture condition. After incubation, bacterial suspensions were centrifuged at 10,000 × g for 15 min. The supernatants were stored at -20°C for further investigation and the bacterial pellets were flash frozen in liquid nitrogen and rapidly stored at -80°C. The next day, total RNA was extracted as previously described [44]. Briefly, the bacterial pellet was resuspended in TE buffer (10 mM Tris-HCl, 1 mM EDTA, pH 8) in 2 mL Graduated Skirted tube with tethered screw cap (BioSpec, USA) containing 600 mg 0.1 mm diameter zirconia/silica beads (Biospec, USA), 1 volume of AquaPhenol™ (pH 4.5) (MP Biomedicals), 1/10 volume of 10% sodium dodecyl sulfate, and 3.5 μL β-mercaptoethanol (PROLABO, France). The lysis of bacterial cells was performed using a FastPrep®-24 instruments (MP Biomedicals) twice for 30 seconds with a speed of 6 m/s. Total RNA was then purified from the bacterial pellet using the Nucleospin® RNA (Macherey Nagel) according to the manufacturer recommendations. To assess DNA contamination, PCR control on each sample was performed using the *tufA* primers (TGGTTGATGACGAAGAGCTG and GCTCTGGTTCCGGAATGTAG). The RNA concentration was measured using a Nanodrop ND-1000 spectrophotometer (Nanodrop technologies, France) and the RNA quality was analyzed using an Agilent 2100 Bioanalyser (Agilent technologies, France). The high quality of each RNA samples was confirmed with 23S/16S rRNA ratio ≈ 2 and RNA Integrity Number ≥ 8.

Enriched fractions of mRNA were prepared using the MicrobExpress™ Bacterial mRNA Purification kit (Ambion) according to the manufacturer instructions. The reduction in 16S and 23S rRNA in mRNA enriched fractions was confirmed using an Agilent 2100 Bioanalyser (Agilent technologies, France).

### RNA Sequencing

RNA-Seq was performed at the GeT-PlaGe core facility, INRA Toulouse, France. RNA-seq libraries were prepared according to Illumina’s protocols using the Illumina TruSeq Stranded mRNA sample prep kit. Briefly, after mRNA enrichment, 200 ng of mRNA were fragmented to generate double stranded cDNA and adapters were ligated. A total of 10 cycles of PCR were applied to amplify libraries. Library quality was assessed using an Advanced Analytical Fragment Analyzer and libraries were quantified by qPCR using the Kapa Library Quantification kit. RNA-seq experiments were performed on an Illumina HiSeq3000 using a paired-end read length of 2x150 bp with the Illumina HiSeq3000 chemistry. A total of 11 to 42 million paired-reads per sample was obtained, except for the sample EDL933_21 (122 million paired-reads) (Additional file 1: Table S2).

### RNA-Seq data analysis and bioinformatics

Raw read files have been stored in ng6 [46] and were checked using fastQC [47]. The sequencing adapters were removed using cutadapt (version 1.8.3, standard parameters) [48]. The reads were then aligned to EDL933 genome (Genbank accession numbers NZ_CP008957.1 and NZ_CP008958.1) [49] using bwa mem (version 0.7.12-r1039, standard options) [50]. Reads were counted using featureCount (version v1.4.5-p1) [51]. Read counts corresponding to the 131 rRNA, tRNA and ncRNA genes were excluded [49]. Differential gene expression was then performed using DESeq2 version 1.12.4 [52] with R version 3.3.2 [53] following the standard workflow. The DESeq2 method internally corrects for library size and uses negative binomial generalized linear models to test for differential expression. All genes with a log2 fold-change (log2FC) in expression greater than 2 and a Benjamini-Hochberg adjusted *p*-value (*q*-value) smaller than 0.05 were reported as differentially expressed. For further analysis, additional gene annotations from older published EDL933 chromosome and plasmid sequences were collected (Genbank accession numbers CP008957.1 and CP008958.1 [49]; AE005174.2 [54] and AF074613.1 [55]). Additional gene annotations were also performed using the Kyoto Encyclopedia of Genes and Genomes (KEGG). Differentially expressed genes were assigned to functional categories of Clusters of Orthologous Groups (COGs) of proteins using blastp against the NCBI COG 2014 database [56]. RNA-seq data have been deposited under SRA accession SRP136076.

Note that the fold change was first calculated by comparing the expression ratio of each gene from a specific DC relative to M9-Glc. This allowed us to identify the genes commonly up-regulated in several DCs. The fold change was also calculated for each gene by directly comparing its expression level between two digestive compartments, in order to identify genes specifically up-regulated in each DC.

### Reverse transcription followed by quantitative PCR (RT-qPCR)

One microgram of each RNA sample (in triplicates) was reverse transcribed using the SuperScript II Reverse Transcriptase kit (Invitrogen) with 3 μg of random primer and 100 units of SuperScript II Rnase H. Quantitative PCR runs were carried out using the Mastercycler ep realplex apparatus (Eppendorf) with 20 ng of cDNA, 0.5 mM of each primer, 10 μL of SYBR Premix Ex Taq mix (Takara Bio Inc.) in a final volume of 20 μL. Amplification conditions were as follows: 95°C for 15 s, 55°C for 15 s, and 72°C for 20 s. The house keeping gene *mdh* was used for normalization of mRNA quantification (Additional file 1: Table S3). The relative RNA quantification was performed using primers designed to specifically amplify fragments of 90 to 200 bp (Additional file 1: Table S3). Triplicate samples were amplified in each case. Results were calculated using the comparative cycle threshold method. The results presented are average from at least duplicate experiments.

### Statistical Analysis

Statistical analyses of the growth and survival of *E. coli* strains in DCs were analysed by the two-way ANOVA with the Bonferroni post-hoc test with *p* < 0.05 considered as significant. For RT-qPCR, Student t-test and the one-way ANOVA with the Tukey post-hoc test were used to identify significant differences in gene expression (*p* < 0.05 was considered as significantly different). The statistical analysis of RNA-seq data is presented above. PCA plot was generated using plotPCA function from DESeq2 package version 1.12.4 and ggplot2 version 2.2.0.

## Additional files

Additional file 1: Supplemental Figures showing the growth of E. coli strains in digestive contents and minimal media and supplemental tables describing the strains used in this study, the RNA-seq mapping assessment, the PCR primers and RT-qPCR results. (DOCX 193 kb)

Additional file 2: Supplemental Tables showing the results of RNAseq statistical analysis of EDL933 incubated in the digestive contents versus minimal medium. (XLSX 557 kb)

Additional file 3:  Supplemental Tables showing the COG classification of differentially expressed genes in EDL933 incubated in digestive contents compared to M9-Glc and genes up-regulated between digestive contents. (DOCX 31 kb)

## Abbreviations

CFU: Colony forming unit
COG: Cluster of orthologous group
DC: Digestive content
EA: Ethanolamine
EHEC: Enterohemorrhagic *Escherichia coli*
GalNAc: N-acetylgalactosamine
GIT: Gastrointestinal tract
GlcNAc: N-acetylglucosamine
HC: Hemorrhagic colitis
HUS: Hemolytic and uremic syndrome
Log2FC: Log2 fold-change
M9-Glc: M9 minimal medium supplemented with glucose
Neu5Ac: N-acetylneuraminic acid
Rif^R^: Rifampicin-resistant
RNA-seq: RNA sequencing
SCFA: Short chain fatty acid
STEC: Shiga toxin producing *Escherichia coli*

## References

[1] Gomes TA, Elias WP, Scaletsky IC, Guth BE, Rodrigues JF, Piazza RM (2016). Diarrheagenic *Escherichia coli*. Braz J Microbiol.

[2] Kaper JB, O'Brien AD. Overview and historical perspectives. Microbiol Spectr. 2014;2(6):EHEC-0028-2014. 10.1128/microbiolspec.EHEC-0028-2014 PMC4290666 25590020

[3] Persad AK, LeJeune JT (2014). Animal reservoirs of shiga toxin producing *Escherichia coli*. Microbiol Spectr.

[4] Hoey DE, Currie C, Else RW, Nutikka A, Lingwood CA, Gally DL (2002). Expression of receptors for verotoxin 1 from *Escherichia coli* O157 on bovine intestinal epithelium. J Med Microbiol..

[5] Pruimboom-Brees IM, Morgan TW, Ackermann MR, Nystrom ED, Samuel JE, Cornick NA (2000). Cattle lack vascular receptors for *Escherichia coli* O157:H7 shiga toxins. Proc Natl Acad Sci U S A.

[6] Saeedi P, Yazdanparast M, Behzadi E, Salmanian AH, Mousavi SL, Nazarian S (2017). A review on strategies for decreasing *E. coli* O157:H7 risk in animals. Microb Pathog.

[7] Chaucheyras-Durand F, Faqir F, Ameilbonne A, Rozand C, Martin C (2010). Fates of acid-resistant and non-acid-resistant shiga toxin-producing *Escherichia coli* strains in ruminant digestive contents in the absence and presence of probiotics. Appl Environ Microbiol.

[8] Chaucheyras-Durand F, Madic J, Doudin F, Martin C (2006). Biotic and abiotic factors influencing *in vitro* growth of *Escherichia coli* O157:H7 in ruminant digestive contents. Appl Environ Microbiol.

[9] de Vaux A, Morrison M, Hutkins RW (2002). Displacement of *Escherichia coli* O157:H7 from rumen medium containing prebiotic sugars. Appl Environ Microbiol.

[10] Grauke LJ, Kudva IT, Yoon JW, Hunt CW, Williams CJ, Hovde CJ (2002). Gastrointestinal tract location of *Escherichia coli* O157:H7 in ruminants. Appl Environ Microbiol.

[11] Keen JE, Laegreid WW, CG C-MK, Durso LM, Bono JL (2010). Distribution of shiga-toxigenic *Escherichia coli* O157 in the gastrointestinal tract of naturally O157-shedding cattle at necropsy. Appl Environ Microbiol.

[12] Kudva IT, Dean-Nystrom EA (2011). Bovine recto-anal junction squamous epithelial (RSE) cell adhesion assay for studying *Escherichia coli* O157 adherence. J Appl Microbiol.

[13] Lim JY, Li J, Sheng H, Besser TE, Potter K, Hovde CJ (2007). *Escherichia coli* O157:H7 colonization at the rectoanal junction of long-duration culture-positive cattle. Appl Environ Microbiol.

[14] Low JC, McKendrick IJ, McKechnie C, Fenlon D, Naylor SW, Currie C (2005). Rectal carriage of enterohemorrhagic *Escherichia coli* O157 in slaughtered cattle. Appl Environ Microbiol.

[15] Naylor SW, Low JC, Besser TE, Mahajan A, Gunn GJ, Pearce MC (2003). Lymphoid follicle-dense mucosa at the terminal rectum is the principal site of colonization of enterohemorrhagic *Escherichia coli* O157:H7 in the bovine host. Infect Immun.

[16] Freter R, Brickner H, Botney M, Cleven D, Aranki A (1983). Mechanisms that control bacterial populations in continuous-flow culture models of mouse large intestinal flora. Infect Immun.

[17] Bertin Y, Chaucheyras-Durand F, Robbe-Masselot C, Durand A, de la Foye A, Harel J (2013). Carbohydrate utilization by enterohaemorrhagic *Escherichia coli* O157:H7 in bovine intestinal content. Environ Microbiol.

[18] Dziva F, van Diemen PM, Stevens MP, Smith AJ, Wallis TS (2004). Identification of *Escherichia coli* O157:H7 genes influencing colonization of the bovine gastrointestinal tract using signature-tagged mutagenesis. Microbiology.

[19] Snider TA, Fabich AJ, Conway T, Clinkenbeard KD (2009). *E. coli* O157:H7 catabolism of intestinal mucin-derived carbohydrates and colonization. Vet Microbiol.

[20] Bertin Y, Girardeau JP, Chaucheyras-Durand F, Lyan B, Pujos-Guillot E, Harel J (2011). Enterohaemorrhagic *Escherichia coli* gains a competitive advantage by using ethanolamine as a nitrogen source in the bovine intestinal content. Environ Microbiol.

[21] Bertin Y, Deval C, de la Foye A, Masson L, Gannon V, Harel J (2014). The gluconeogenesis pathway is involved in maintenance of enterohaemorrhagic *Escherichia coli* O157:H7 in bovine intestinal content. PLoS One.

[22] Unden G, Strecker A, Kleefeld A, Kim OB. C_4_-dicarboxylate utilization in aerobic and anaerobic growth. EcoSal Plus. 2016;7(1). ESP-0021-2015. 10.1128/ecosalplus.esp-0021-2015 PMC11575717 27415771

[23] Deplancke B, Gaskins HR (2001). Microbial modulation of innate defense: goblet cells and the intestinal mucus layer. Am J Clin Nutr.

[24] Sicard JF, Le Bihan G, Vogeleer P, Jacques M, Harel J (2017). Interactions of intestinal bacteria with components of the intestinal mucus. Front Cell Infect Microbiol.

[25] Montagne L, Toullec R, Lalles JP (2000). Calf intestinal mucin: isolation, partial characterization, and measurement in ileal digesta with an enzyme-linked immunosorbent assay. J Dairy Sci.

[26] Aperce CC, Heidenreich JM, Drouillard JS (2014). Capacity of the bovine intestinal mucus and its components to support growth of *Escherichia coli* O157:H7. Animal.

[27] Chang DE, Smalley DJ, Tucker DL, Leatham MP, Norris WE, Stevenson SJ (2004). Carbon nutrition of *Escherichia coli* in the mouse intestine. Proc Natl Acad Sci U S A.

[28] Fabich AJ, Jones SA, Chowdhury FZ, Cernosek A, Anderson A, Smalley D (2008). Comparison of carbon nutrition for pathogenic and commensal *Escherichia coli* strains in the mouse intestine. Infect Immun.

[29] Bertin Y, Habouzit C, Dunière L, Laurier M, Durand A, Duchez D (2017). *Lactobacillus reuteri* suppresses *E. coli* O157:H7 in bovine ruminal fluid: Toward a pre-slaughter strategy to improve food safety?. PLoS One.

[30] McSweeney CS, Mackie R (2012). Micro-organisms and ruminant digestion: state of knowledge, trends and future prospects. Food and Agriculture Organization of the United Nation Background study paper N°61.

[31] Jones SA, Jorgensen M, Chowdhury FZ, Rodgers R, Hartline J, Leatham MP (2008). Glycogen and maltose utilization by *Escherichia coli* O157:H7 in the mouse intestine. Infect Immun.

[32] Leatham MP, Banerjee S, Autieri SM, Mercado-Lubo R, Conway T, Cohen PS (2009). Precolonized human commensal *Escherichia coli* strains serve as a barrier to *E. coli* O157:H7 growth in the streptomycin-treated mouse intestine. Infect Immun.

[33] Saraoui T, Parayre S, Guernec G, Loux V, Montfort J, Le Cam A (2013). A unique *in vivo* experimental approach reveals metabolic adaptation of the probiotic *Propionibacterium freudenreichii* to the colon environment. BMC Genomics.

[34] Matheron C, Delort AM, Gaudet G, Forano E, Liptaj T (1998). 13C and 1H nuclear magnetic resonance study of glycogen futile cycling in strains of the genus *Fibrobacter*. Appl Environ Microbiol.

[35] Pacheco AR, Curtis MM, Ritchie JM, Munera D, Waldor MK, Moreira CG (2012). Fucose sensing regulates bacterial intestinal colonization. Nature.

[36] Miranda RL, Conway T, Leatham MP, Chang DE, Norris WE, Allen JH (2004). Glycolytic and gluconeogenic growth of *Escherichia coli* O157:H7 (EDL933) and *E. coli* K-12 (MG1655) in the mouse intestine. Infect Immun.

[37] Kendall MM, Gruber CC, Parker CT, Sperandio V. Ethanolamine controls expression of genes encoding components involved in interkingdom signaling and virulence in enterohemorrhagic *Escherichia coli* O157:H7. MBio. 2012;3(3). 10.1128/mBio.00050-12 PMC3372972 22589288

[38] Espey MG (2013). Role of oxygen gradients in shaping redox relationships between the human intestine and its microbiota. Free Radic Biol Med.

[39] He G, Shankar RA, Chzhan M, Samouilov A, Kuppusamy P, Zweier JL (1999). Noninvasive measurement of anatomic structure and intraluminal oxygenation in the gastrointestinal tract of living mice with spatial and spectral EPR imaging. Proc Natl Acad Sci U S A.

[40] Jones SA, Chowdhury FZ, Fabich AJ, Anderson A, Schreiner DM, House AL (2007). Respiration of *Escherichia coli* in the mouse intestine. Infect Immun.

[41] Jones SA, Gibson T, Maltby RC, Chowdhury FZ, Stewart V, Cohen PS (2011). Anaerobic respiration of *Escherichia coli* in the mouse intestine. Infect Immun.

[42] Unden G, Bongaerts J (1997). Alternative respiratory pathways of *Escherichia coli*: energetics and transcriptional regulation in response to electron acceptors. Biochim Biophys Acta.

[43] Bertin Y, Segura A, Jubelin G, Dunière L, Durand A, Forano E. Aspartate metabolism is involved in the maintenance of enterohaemorrhagic *Escherichia coli* O157:H7 in bovine intestinal content. Environ Microbiol. 2018. 10.1111/1462-2920.14380. 10.1111/1462-2920.14380 30109758

[44] Segura A, Auffret P, Bibbal D, Bertoni M, Durand A, Jubelin G (2018). Factors involved in the persistence of a shiga toxin-producing *Escherichia coli* O157:H7 strain in bovine feces and gastro-intestinal content. Front Microbiol.

[45] Sambrook J, Fritsch EF, Maniatis T, Ausubel FM, Brent M, Kingston RE, Moore JG (1989). Current protocols in molecular biology. Molecular cloning: A laboratory manual. 1 and 2. Seconde ed.

[46] Mariette J, Escudie F, Allias N, Salin G, Noirot C, Thomas S (2012). NG6: Integrated next generation sequencing storage and processing environment. BMC Genomics.

[47] Andrews S. FastQC: a quality control tool for high throughput sequence data. http://www.bioinformatics.babraham.ac.uk/projects/fastqc/ (2010). Accessed 10 Nov 2016.

[48] Martin M (2011). Cutadapt removes adapter sequences from high-throughput sequencing reads. EMBnet J.

[49] Latif H, Li HJ, Charusanti P, Palsson BO, Aziz RK (2014). A gapless, unambiguous genome sequence of the enterohemorrhagic *Escherichia coli* O157:H7 strain EDL933. Genome Announc.

[50] Li H, Durbin R (2009). Fast and accurate short read alignment with Burrows-Wheeler transform. Bioinformatics.

[51] Liao Y, Smyth GK, Shi W (2014). featureCounts: an efficient general purpose program for assigning sequence reads to genomic features. Bioinformatics.

[52] Love MI, Huber W, Anders S (2014). Moderated estimation of fold change and dispersion for RNA-seq data with DESeq2. Genome Biol.

[53] R Core Team, R: A language and environment for statistical computing The R Project for Statistical Computing, Vienna, Austria. https//www.r-project.org (2016). Accessed 20 Dec 2016.

[54] Perna NT, Plunkett G, Burland V, Mau B, Glasner JD, Rose DJ (2001). Genome sequence of enterohaemorrhagic *Escherichia coli* O157:H7. Nature.

[55] Burland V, Shao Y, Perna NT, Plunkett G, Sofia HJ, Blattner FR (1998). The complete DNA sequence and analysis of the large virulence plasmid of *Escherichia coli* O157:H7. Nucleic Acids Res.

[56] Geer LY, Marchler-Bauer A, Geer RC, Han L, He J, He S (2010). The NCBI BioSystems database. Nucleic Acids Res.

