# Supplementary material for: Transcriptomic analysis reveals specific metabolic pathways of enterohemorrhagic Escherichia coli O157:H7 in bovine digestive contents
Source: BMC Genomics. 2018 Oct 23;19:766. doi: 10.1186/s12864-018-5167-y (PMC6199705; doi:10.1186/s12864-018-5167-y)
Supplement: Supplementary file 1 — Supplemental Figures showing the growth of E. coli strains in digestive contents and minimal media and supplemental tables describing the strains used in this study, the RNA-seq mapping assessment, the PCR primers and RT-qPCR results. (DOCX 193 kb) [file 12864_2018_5167_MOESM1_ESM.docx]

**Additional file 1**

**Fig. S1 Growth curves of EHEC EDL933 incubated in filtered digestive contents and M9-Glc.**

Incubations were performed at 39°C under growth conditions described in the Materials and Methods section. A. Rumen content, B. Small intestine and rectum contents, C. M9-Glc

B

C

A

**Fig. S2 Growth curves of different *E. coli* strains incubated in unfiltered bovine digestive contents.**

A) O157:H7 EHEC strain Sakai Rif^R^, B) O157:H7 bovine STEC strain NV95 Rif^R^, C) Bovine commensal *E. coli* strain BG1 Rif^R^


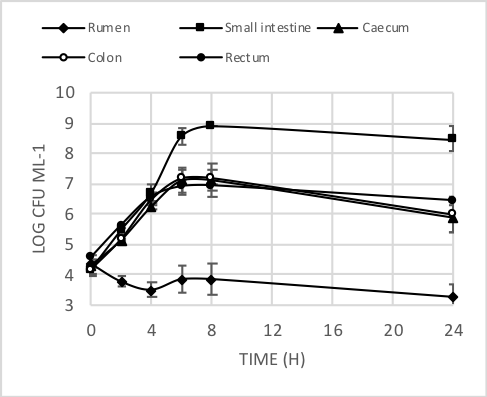


A


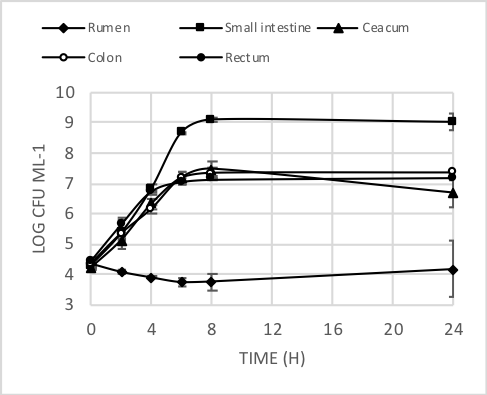


B


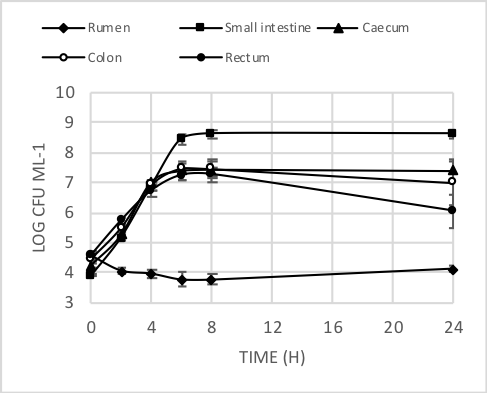


C

**Table S1 Bacterial strains**

| Strain | Serotype | Origin | Genetic characteristic | | | Reference |
| --- | --- | --- | --- | --- | --- | --- |
|  |  |  | *eae* | *stx1* | *stx2* |  |
| EDL933 | O157:H7 | HC/outbreak | + | + | + | [54, 57] |
| EDL933 Rif^R^ | O157:H7 |  | + | + | + | [17] |
| Sakai | O157:H7 | HUS/outbreak | + | + | + | [58] |
| Sakai Rif^R^ | O157:H7 |  | + | + | + | This work |
| NV95 | O157:H7 | Bovine feces | + | + | + | [59] |
| NV95 Rif^R^ | O157:H7 |  | + | + | + | This work |
| BG1 | O159:H21 | Bovine small intestine | - | - | - | [20, 60] |
| BG1 Rif^R^ | O159:H21 |  | - | - | - | [17] |

57. Wells JG, Davis BR, Wachsmuth IK, Riley LW, Remis RS, Sokolow R, et al. Laboratory investigation of hemorrhagic colitis outbreaks associated with a rare *Escherichia coli* serotype. J Clin Microbiol. 1983;18(3):512-20.

58. Watanabe Y, Ozasa K, Mermin JH, Griffin PM, Masuda K, Imasuku S, et al. Factory outbreak of *Escherichia coli* O157:H7 infection in Japan. Emerg Infect Dis. 1999;5(3):424-8.

59. Pradel N, Livrelli V, De Champs C, Palcoux JB, Reynaud A, Scheutz F, et al. Prevalence and characterization of Shiga toxin-producing *Escherichia coli* isolated from cattle, food, and children during a one-year prospective study in France. J Clin Microbiol. 2000;38(3):1023-31.

60. Segura A, Auffret P, Klopp C, Bertin Y, Forano E. Draft genome sequence and characterization of commensal *Escherichia coli* strain BG1 isolated from bovine gastro-intestinal tract. Standards in genomic sciences. 2017;12:61.

**Table S2 RNA-seq mapping assessment**

| **RNA-seq**  **Sample ID** | **Digestive contents**  **Growth phase** | **Total reads generated** | **Reads after trimming** | **Mapped reads to EDL933 genome (% mapped)** | |
| --- | --- | --- | --- | --- | --- |
| EDL933_1 | Rumen | 42,939,146 | 42,923,152 | 42,672,416 | (99.42) |
| EDL933_2 |  | 34,986,450 | 34,979,296 | 34,867,530 | (99.68) |
| EDL933_3 |  | 28,030,862 | 28,025,264 | 27,963,762 | (99.78) |
| EDL933_4 | Small intestine  (Mid-exponential growth phase) | 37,901,152 | 37,893,572 | 37,733656 | (99.58) |
| EDL933_5 |  | 34,565,456 | 34,553,404 | 34,471,754 | (99.76) |
| EDL933_6 |  | 30,448,202 | 30,441,850 | 30,343,730 | (99.68) |
| EDL933_7 | Small intestine  (Late-exponential growth phase) | 34,095,408 | 30,088,580 | 33,990,068 | (99.71) |
| EDL933_8 |  | 16,921,418 | 16,913,888 | 16,888,432 | (99.85) |
| EDL933_9 |  | 35,578,220 | 35,295,296 | 35,143,248 | (98.57) |
| EDL933_10 | Rectum  (Mid-exponential growth phase) | 31,104,402 | 31,036,286 | 30,924,190 | (99.64) |
| EDL933_11 |  | 18,983,622 | 18,557,648 | 18,464,044 | (97.50) |
| EDL933_12 |  | 15,122,874 | 14,782,904 | 14,685,124 | (97.34) |
| EDL933_13 | Rectum  (Late-exponential growth phase) | 14,311,528 | 14,089,908 | 14,028,484 | (98.56) |
| EDL933_14 |  | 11,285,778 | 11,096,106 | 11,039,860 | (97.49) |
| EDL933_15 |  | 31,898,954 | 31,870332 | 31,760,648 | (99.66) |
| EDL933_16 | M9-Glc  (Mid-exponential growth phase) | 35,645,614 | 35,630,108 | 35,510,990 | (99.67) |
| EDL933_17 |  | 35,761,134 | 35,751,146 | 35,547,424 | (99.43) |
| EDL933_18 |  | 21,499,258 | 21,494,146 | 21,404,684 | (99.58) |
| EDL933_19 | M9-Glc  (Late-exponential growth phase) | 23,011,282 | 23,006,292 | 22,933,804 | (99.68) |
| EDL933_20 |  | 21,878,430 | 21,873,106 | 21,802,224 | (99.68) |
| EDL933_21 |  | 121,921,678 | 121,874,896 | 121,457,454 | (99.66) |
| **Total** |  | **677,890,868** | **676,177,170** | **673,633,526** |  |

**Table S3 Primers used for RT-qPCR**

| Gene tag | Gene | Forward | Reverse | Reference |
| --- | --- | --- | --- | --- |
| Z4488 | *agaF* | TTTATCGCCATCGATTTTCC | CAGCACCATCTCCAGTAGCA | [17] |
| Z0070 | *araA* | GAAACAGCTTCCTGGTCTGG | GGTCATTGCCTTTCTCGAAG | This study |
| Z5245 | *asnA* | AACCGCTTACATTGCCAAAC | CACAGCCCGACAAGTTATCC | This study |
| Z5744 | *aspA* | CTTGCTCTCTTCTGGTCCA | AACATAGCCTGGCCAATGAC | [21] |
| Z4942 | *dctA* | GTGGTGCTGGTATTGGGTTC | GAGTAGCCTGTCGGGATGAC | [21] |
| Z5743 | *dcuA* | CTGGTTGAAAAACCGCTGAT | TCGATGTTGTTGGAAACGAA | This study |
| Z5725 | *dcuB* | GCCATCTTGAGTTCCTCGAC | ACTCACGGTTTTTCGGTACG | This study |
| Z3706 | *eutB* | GTGCGGGCTTAGAAGATCAC | GAATGCGGTGGTCTGGTAGT | [20] |
| Z2236 | *fdnG* | CGATATCCCTGACTTCGGC | TTGTTCATACAGACGAACCAC | This study |
| Z5762 | *frdA* | CGGTATCCTGATGACCGAAG | GTGGAGATGGTGTTGCCTTT | This study |
| Z3425 | *fruA* | GCCATGCGAAACCTTACACT | CACGGGTTTCAACTTTCACC | This study |
| Z4117 | *fucA* | AGGATGGGATGCTGATTACG | CGGTCGGTTAAGAATGGAAA | [17] |
| Z2615 | *fumA* | AGTGAATACCGGCACCAATC | GCGTCAGTAACGCTTTGGTT | This study |
| Z0927 | *galK* | AGTTCTTCCGCTTCACTGGA | GAGCGACAGTCAATCAGCAA | [17] |
| Z3499 | *glpA* | TGAAGTGCAGTACGCGGTAG | CCTTTCCAGCGTTCGTTAAG | This study |
| Z4786 | *glpD* | CATCAATGGTGCTGGTATCG | TTCAGCAGCACTTCACGTTC | This study |
| Z5472 | *glpF* | TACCATTGCATTGTGGCT | AAAGTGCCTGCCAGATCAAC | This study |
| Z3498 | *glpT* | TAACGAAAAAGCGGAACAGG | AAGAAGTAGGCCCAGGAGGA | This study |
| Z4805 | *gntK* | TTCTTGATGGCGATTTCCTC | TACCTTCACGCAGCAAGTCA | This study |
| Z1390 | *hyaB* | GGCGTTCGTTGAACGTATCT | CATCCAACACATCGATCCAG | This study |
| Z4595 | *mdh* | GGAACTGAAAGGCAAACAGC | GCGTTCTGGATACGTTTGGT | This study |
| Z4875 | *z4875* | GAACAGATCATCCGCCAGTT | CATGTTTATGGTCCGTGTGC | This study |
| Z0826 | *nagE* | CAGGTTCCTGACGAAGCATT | CTTTACCTTCCAGCGCTACG | [17] |
| Z4583 | *nanA* | CCGTCACGCCGTTCTACTAT | GATCGCCAGAGGTCTGTTTC | [17] |
| Z3463 | *napA* | ACGGTAAAGACCGTTTGACG | CGGCATAACCTTCCCAGATA | This study |
| Z2001 | *narG* | GAAGGCGCTGCATAGCGATC | CGAGAAACCAGCAACACGG | This study |
| Z4758 | *pckA* | GTACGCAGAAGTGCTGGTGA | GGCAGAGTGAAGGTTTCTGC | This study |
| Z5250 | *rbsA* | GAAATTCTTGGCGTCTCTGG | GCCCAACACTAAACCGTCAC | This study |
| Z2857 | *sdaA* | ATGCTGGTTTCCAGCGATAA | TATAGATGTCCGGGCTGACC | This study |
| Z0877 | *sdh*A | GGGTCGGACTATATCGGTGA | GCCGAAGTTTTTCGACTGAC | This study |
| Z0882 | *sucC* | GACCTGATCGCTGACGGTAT | CTGCATCCGTCAGACCTTTT | This study |
| Z4467 | *tdcD* | GGAAGGCTTGATGATGGGTA | GCAATTCGGTGAACAAAGGT | [21] |
| Z5203 | *tnaA* | TACACCATTCCGACTCACCA | CCGTATCGAAGGCTTCTT | This study |

**Table S4 Quantification of expression (Log2 fold-change) of selected genes in EDL933 incubated in rumen, small intestine and rectum contents relative to M9-Glc by**

**RT-qPCR and comparison with RNA-seq data.**

| **Gene Tag** | **Gene** | **Log2 fold-change** | | | | | | | | | | | | | | | | |  |
| --- | --- | --- | --- | --- | --- | --- | --- | --- | --- | --- | --- | --- | --- | --- | --- | --- | --- | --- | --- |
|  |  | **3 hours of incubation** | | | | | | **6 hours of incubation** | | | | | | | | | | |  |
|  |  | **Small intestine** | | | **Rectum** | | | **Small intestine** | | | | **Rectum** | | | | **Rumen** | | |  |
|  |  | RNA-seq | RT-qPCR | *p*-value^d^ | RNA-seq | RT-qPCR | *p*-value^d^ | RNA-seq | | RT-qPCR | *p*-value^d^ | RNA-seq | | RT-qPCR | *p*-value^d^ | RNA-seq | RT-qPCR | *p*-value^d^ |  |
| Z4488 | *agaF* | NDE | 0.19 | 0.7976 | 3.23 | 2.68* | 0.0013 | | NDE | 1.55 | 0.0423 | | 3.20 | 2.67 | 0.0204 | NDE | 2.95 | 0.0060 | |
| Z0070 | *araA* | 2.79 | 1.92 | 0.0628 | NDE | 1.98 | 0.0338 | | 3.14 | 2.00 | 0.0258 | | 2.22 | 2.23 | 0.0189 | NDE | 2.94 | 0.0066 | |
| Z5245 | *asnA* | NDE | -0.83 | 0.2554 | 2.68 | 3.37* | 0.0263 | | 3.11 | 3.80 | 0.0056 | | 2.84 | 2.53 | 0.0004 | 3.05 | 2.63 | 0.0095 | |
| Z5744 | *aspA* | 6.08 | 5.96 | 0.0002 | 5.03 | 5.08 | < 0.0001 | | 5.34 | 5.44^a^ | 0.0019 | | 2.84 | 1.92^b^ | 0.0113 | 4.46 | 4.59^a^ | 0.0008 | |
| Z4942 | *dctA* | 4.13 | 3.22 | 0.0059 | 3.91 | 3.06 | 0.0034 | | 3.35 | 2.28 | 0.0035 | | 3.65 | 2.20 | 0.0033 | 2.11 | 2.42 | 0.0006 | |
| Z5743 | *dcuA* | 3.21 | 1.90 | 0.0017 | 2.51 | 1.98 | 0.0003 | | 2.12 | 2.96 | 0.0016 | | NDE | 0.48 | 0.4782 | NDE | 3.02 | 0.0027 | |
| Z5725 | *dcuB* | 2.98 | 1.08 | 0.0793 | 3.49 | 3.21* | < 0.0001 | | 3.26 | 2.87 | 0.0266 | | NDE | 2.23 | 0.0282 | 2.11 | 3.10 | 0.0013 | |
| Z3706 | *eutB* | NDE | 0.68 | 0.1406 | NDE | 2.24* | 0.0009 | | 3.68 | 5.11^b^ | < 0.0001 | | 3.12 | 3.68^ab^ | 0.0032 | 4.02 | 2.01^a^ | 0.0001 | |
| Z2236 | *fdnG* | NDE | 1.94 | 0.1353 | NDE | 1.44 | 0.0113 | | 2.73 | 1.08^b^ | 0.0100 | | 3.48 | 1.81^ab^ | 0.0003 | 2.06 | 2.73^a^ | < 0.0001 | |
| Z5762 | *frdA* | 4.43 | 2.92 | 0.0016 | 2.24 | 2.27 | 0.0002 | | 4.70 | 3.71^a^ | 0.0014 | | 2.02 | 1.91^b^ | 0.0049 | 4.03 | 4.64^c^ | < 0.0001 | |
| Z3425 | *fruA* | 2.95 | 1.54 | 0.1903 | 2.44 | 2.46 | 0.0076 | | NDE | 1.68 | 0.0310 | | NDE | 0.76 | 0.0472 | NDE | 1.75 | 0.0175 | |
| Z4117 | *fucA* | 3.99 | 1.70 | 0.0649 | 4.05 | 2.44* | 0.0047 | | 3.45 | 1.10^b^ | 0.0049 | | 4.24 | 3.05^a^ | 0.0478 | 2.69 | 2.61^a^ | 0.0010 | |
| Z2615 | *fumA* | 3.90 | 2.63 | 0.0002 | 3.35 | 3.12 | 0.0010 | | 3.50 | 2.06^a^ | 0.0217 | | 3.53 | 2.68^a^ | 0.0008 | 2.50 | 3.92^b^ | 0.0047 | |
| Z0927 | *galK* | 2.55 | -0.05 | 0.8888 | 3.50 | 1.22 | 0.0969 | | NDE | 0.32 | 0.6222 | | NDE | -0.76 | 0.3707 | NDE | 0.32 | 0.6691 | |
| Z3499 | *glpA* | 4.13 | 2.28 | 0.0141 | 2.50 | 1.10 | 0.0037 | | 5.21 | 5.61 | < 0.0001 | | 3.42 | 1.84 | 0.0002 | 4.15 | 2.14 | 0.0004 | |
| Z4786 | *glpD* | 3.62 | 3.31 | 0.0095 | 2.65 | 2.11 | 0.0381 | | 4,98 | 6.28^a^ | 0.0006 | | 4.75 | 4.53^b^ | 0.0002 | 4.23 | 3.31^c^ | 0.0033 | |
| Z5472 | *glpF* | 4.02 | 2.20 | 0.0414 | 3.66 | 2.67 | 0.0021 | | 4.02 | 3.42 | < 0.0001 | | 5.33 | 4.01 | 0.0004 | 2.63 | 2.92 | 0.0007 | |
| Z3498 | *glpT* | 5.65 | 4.69 | 0.0019 | 3.47 | 2.99 | 0.0011 | | 5.44 | 4.86 | 0.0366 | | 3.71 | 3.29 | 0.0030 | 4.05 | 3.29 | 0.0143 | |
| Z4805 | *gntK* | NDE | -1.10 | 0.5351 | 2.44 | 0.32 | 0.6226 | | NDE | 1.74 | 0.0425 | | NDE | 1.43 | 0.0510 | NDE | 2.83 | 0.0117 | |
| Z1390 | *hyaB* | 2.58 | 4.00 | 0.0021 | 2.48 | 6.34 | 0.0008 | | 6.32 | 6.45^a^ | < 0.0001 | | 2.72 | 2.16^b^ | 0.0015 | 5.69 | 5.46^a^ | < 0.0001 | |
| Z4875 | *z4875* | 2.44 | 0.96 | 0.0329 | NDE | 2.28 | 0.0023 | | 3.84 | 2.28^a^ | 0.0001 | | 2.36 | 1.66^a^ | 0.0035 | 3.61 | 3.61^b^ | < 0.0001 | |
| Z0826 | *nagE* | 3.31 | 2.63 | 0.0170 | 4.11 | 4.69 | < 0.0001 | | NDE | 1.36 | 0.0138 | | 2.25 | 1.41 | 0.0510 | NDE | 1.89 | 0.0054 | |
| Z4583 | *nanA* | 7.79 | 7.13* | 0.0008 | 5.14 | 4.12 | 0.0005 | | 3.55 | 2.96 | 0.0254 | | 5.90 | 5.67 | 0.0011 | NDE | 3.23 | 0.0030 | |
| Z3463 | *napA* | 2.79 | 0.63 | 0.3426 | NDE | 2.10 | 0.0733 | | 2.66 | 1.48 | 0.0473 | | NDE | 2.14 | 0.0469 | 2.77 | 2.77 | 0.0139 | |
| Z2001 | *narG* | NDE | -0.57 | 0.4439 | NDE | 1.69 | 0.1563 | | 4.50 | 2.80^ab^ | 0.0027 | | NDE | 1.22^b^ | 0.1190 | 4.97 | 4.77^a^ | 0.0007 | |
| Z4758 | *pckA* | 3.54 | 2.74 | 0.0007 | 2.60 | 2.01 | 0.0067 | | 2.86 | 3.26 | 0.0003 | | 3.44 | 2.82 | 0.0038 | NDE | 2.37 | 0.0024 | |
| Z5250 | *rbsA* | 6.24 | 3.04 | 0.0049 | 2.09 | 3.63 | 0.0009 | | 2.84 | 2.54 | 0.0022 | | 5.80 | 3.13 | 0.0003 | 4.60 | 3.80 | 0.0044 | |
| Z2857 | *sdaA* | 2.18 | 1.06 | 0.0723 | NDE | 1.09 | 0.0028 | | 2.46 | 1.95 | 0.0058 | | 2.47 | 2.05 | 0.0122 | 2.25 | 2.78 | 0.0029 | |
| Z0877 | *sdh*A | NDE | -0.22 | 0.7970 | NDE | 0.85 | 0.1199 | | NDE | 0.76^ab^ | 0.0070 | | 2.12 | 1.75^b^ | 0.0112 | NDE | -0.25^a^ | 0.5901 | |
| Z0882 | *sucC* | NDE | -0.50 | 0.4966 | NDE | -0.64 | 0.2931 | | NDE | 1.70^ab^ | 0.0008 | | 2.93 | 2.37^b^ | 0.0012 | NDE | 0.37^a^ | 0.3041 | |
| Z4467 | *tdcD* | 5.22 | 2.63 | 0.0329 | NDE | 2.19 | 0.0023 | | 5.89 | 3.85 | < 0.0001 | | 2.29 | 3.31 | 0.0282 | 4.22 | 2.59 | 0.0003 | |
| Z5203 | *tnaA* | 3.16 | 1.77 | 0.0133 | NDE | 2.44 | 0.0056 | | 4.73 | 4.38 | 0.0010 | | 2.48 | 2.51 | 0.0251 | 3.62 | 2.63 | 0.0004 | |

NDE: not differentially expressed

* Log2FC was significantly different between small intestine and rectum contents during the mid-exponential growth phase (3 hours of incubation) (Student t-test, *p* < 0.05)

^a, b, c^ Log2FC was significantly different between DC at 6 hours of incubation (One-way ANOVA with the Tukey post-hoc test, *p* < 0.05)

^d^ *p*-value was determined by a Student t-test comparing the level of gene expression obtained by RT-qPCR and observed between DC and M9-Glc. *p* < 0.05 was considered as significantly different
