# Supplementary material for: Transcriptomic analysis reveals specific metabolic pathways of enterohemorrhagic Escherichia coli O157:H7 in bovine digestive contents
Source: BMC Genomics. 2018 Oct 23;19:766. doi: 10.1186/s12864-018-5167-y (PMC6199705; doi:10.1186/s12864-018-5167-y)
Supplement: Supplementary file 3 — Supplemental Tables showing the COG classification of differentially expressed genes in EDL933 incubated in digestive contents compared to M9-Glc and genes up-regulated between digestive contents. (DOCX 31 kb) [file 12864_2018_5167_MOESM3_ESM.docx]

**Additional file 3**

**Table S6. COG classification of differentially expressed genes (log2FC > │2│; *q* < 0.05) in EDL933 incubated in rumen, small intestine and rectum contents during each growth phases compared to M9-Glc**

| **COG** | **Fonction** | **Number of gene** | **% number of gene** |
| --- | --- | --- | --- |
| J | Translation, ribosomal structure and biogenesis | 54 | 1.8 |
| K | Transcription | 176 | 5.7 |
| L | Replication, recombination and repair | 23 | 0.7 |
| D | Cell cycle control, cell division, chromosome partitioning | 2 | 0.1 |
| V | Defense mechanisms | 39 | 1.3 |
| T | Signal transduction mechanisms | 145 | 4.7 |
| M | Cell wall/membrane/envelope biogenesis | 107 | 3.5 |
| N | Cell motility | 91 | 3.0 |
| W | Extracellular structures | 15 | 0.5 |
| U | Intracellular trafficking, secretion, and vesicular transport | 36 | 1.2 |
| O | Posttranslational modification, protein turnover, chaperones | 104 | 3.4 |
| X | Mobilome: prophages, transposons | 17 | 0.6 |
| C | Energy production and conversion | 321 | 10.4 |
| G | Carbohydrate transport and metabolism | 333 | 10.8 |
| E | Amino acid transport and metabolism | 467 | 15.1 |
| F | Nucleotide transport and metabolism | 83 | 2.7 |
| H | Coenzyme transport and metabolism | 138 | 4.5 |
| I | Lipid transport and metabolism | 55 | 1.8 |
| P | Inorganic ion transport and metabolism | 318 | 10.3 |
| Q | Secondary metabolites biosynthesis, transport and catabolism | 65 | 2.1 |
| R | General function prediction only | 194 | 6.3 |
| NA | Unknown, unclassified | 705 | 22.9 |

NA: not assigned

**Table S7. COG classification of up-regulated genes in EHEC EDL933 incubated for 6h in filtered rumen, small intestine and rectum contents with a log2FC > 2 compared to M9-Glc**

| **Gene tag** | **Gene** | **Gene product** |
| --- | --- | --- |
| **C: Energy production and conversion** | | |
| Z2315 | *acpD* | FMN-dependent NADH-azoreductase |
| Z4942 | *dctA* | Aerobic C_4_-dicarboxylate transporter |
| Z3703 | *eutK* | Ethanolamine utilization (EutK protein) |
| Z2236 | *fdnG* | Formate dehydrogenase (α subunit) |
| Z5435 | *fdoH* | Formate dehydrogenase-I (iron-sulfur subunit) |
| Z5434 | *fdoI* | Formate dehydrogenase-I (γ subunit) |
| Z5762 | *frdA* | Fumarate reductase (flavoprotein subunit) |
| Z4116 | *fucO* | Lactaldehyde reductase |
| Z2615 | *fumA* | Fumarase |
| Z3499 | *glpA* | Glycerol 3-phosphate dehydrogenase (a subunit) |
| Z4786 | *glpD* | Glycerol 3-phosphate dehydrogenase |
| Z5471 | *glpK* | Glycerol kinase |
| Z1389 | *hyaA* | Hydrogenase I (small subunit) |
| Z1390 | *hyaB* | Hydrogenase I (large subunit) |
| Z1391 | *hyaC* | Hydrogenase I (B-type cytochrome subunit) |
| Z1392 | *hyaD* | Hydrogenase 1 (maturation protease) |
| Z2668 | *nemA* | N-ethylmaleimide reductase |
| Z5671 | *nrfC* | Nitrite reductase (formate-dependent) |
| Z4467 | *tdcD* | Propionate kinase |
| Z4466 | *tdcE* | Formate acetyltransferase |
| **G: Carbohydrate transport and metabolism** | | |
| Z4487 | *agaE* | PTS N-acetylgalactosamine transporter (IID subunit) |
| Z4485 | *agaV* | PTS N-acetylgalactosamine transporter (IIB subunit) |
| Z4486 | *agaW* | PTS N-acetylgalactosamine transporter (IIC subunit) |
| Z4491 | *agaY* | Tagatose-1,6-bisphosphate aldolase (GatY subunit) |
| Z4484 | *agaZ* | Tagatose-1,6-bisphosphate aldolase (GatZ subunit) |
| Z4278 | *cmtB* | Mannitol-specific IIA component |
| Z4117 | *fucA* | L-fuculose-phosphate aldolase |
| Z5472 | *glpF* | Glycerol uptake facilitator protein |
| Z3498 | *glpT* | Glycerol 3-phosphate transporter |
| Z4875 | *z4875* | Galactitol-specific IIA component |
| Z4876 | *z4876* | Galactitol-specific IIB component |
| Z4879 | *z4879* | Phosphocarrier protein |
| Z3394 | *z3394* | MFS transporter |
| Z5250 | *rbsA* | Ribose transport system ATP-binding protein |
| Z5251 | *rbsC* | Ribose transport system permease protein |
| Z5249 | *rbsD* | D-Ribose pyranase |
| Z4990 | *xylA* | Xylose isomerase |
| Z4991 | *xylF* | Xylose transport system substrate-binding protein |
| Z2813 | *ydjK* | MFS transporter |
| Z3431 | *yeiQ* | Putative oxidoreductase |
| **E: Amino acid transport and metabolism** | | |
| Z5245 | *asnA* | Aspartate ammonia-ligase |
| Z5744 | *aspA* | Aspartate ammonia-lyase |
| Z3707 | *eutA* | Ethanolamine utilization (EutA protein) |
| Z3706 | *eutB* | Ethanolamine ammonia-lyase (large subunit) |
| Z3705 | *eutC* | Ethanolamine ammonia-lyase (small subunit) |
| Z3704 | *eutL* | Ethanolamine utilization (EutL protein) |
| Z3717 | *eutP* | Ethanolamine utilization (EutP protein) |
| Z3718 | *eutS* | Ethanolamine utilization (EutS protein) |
| Z3500 | *glpB* | Glycerol 3-phosphate dehydrogenase (b subunit) |
| Z3401 | *preT* | Dihydropyrimidine dehydrogenase (PreT subunit) |
| Z2857 | *sdaA* | L-serine dehydratase |
| Z5203 | *tnaA* | Tryptophanase |

**Table S8. Genes up-regulated in rectum content vs small intestine or vs rumen content**

| **Gene tag** | **Gene** | | | **Gene product** | **3 hours incubation 6 hours incubation** | | | | |  |  |  |  |
| --- | --- | --- | --- | --- | --- | --- | --- | --- | --- | --- | --- | --- | --- |
|  |  |  |  |  | **Rectum/Small intestine** | | **Rectum/Small intestine** | | **Rectum/Rumen** | | |  |  |
|  |  |  |  |  | **Log2FC** | ***q*-value** | **Log2FC** | ***q*-value** | **Log2FC** | | ***q*-value** |  |  |
| Z4492 | | | *agaB* | PTS system galactosamine-specific IIB component | | 2.58 | 8.59E-05 | NDE |  | NDE | |  |  |
| Z4493 | | | *agaC* | PTS system galactosamine-specific IIC component | | 2.20 | 6.33E-04 | NDE |  | NDE | |  |  |
| Z4487 | | | *agaE* | PTS system N-acetylgalactosamine-specific IID component | | NDE |  | NDE |  | NDE | |  |  |
| Z4488 | | | *agaF* | PTS system N-acetylgalactosamine-specific IIA component | | NDE |  | NDE |  | NDE | |  |  |
| Z4490 | | | *agaS* | Aldose isomerase | | 4.02 | 1.54E-12 | NDE |  |  | |  |  |
| Z4485 | | | *agaV* | PTS system N-acetylgalactosamine-specific IIB component | | NDE |  | NDE |  | NDE | |  |  |
| Z4486 | | | *agaW* | PTS system N-acetylgalactosamine-specific IIC component | | NDE |  | NDE |  | NDE | |  |  |
| Z4491 | | | *agaY* | Tagatose 1,6-diphosphate aldolase (GatY/KbaY subunit) | | 4.20 | 3.78E-11 | NDE |  | NDE | |  |  |
| Z4484 | | | *agaZ* | Tagatose 1,6-diphosphate aldolase (GatZ/KbaZ subunit) | | 4.55 | 3.15E-11 | NDE |  | NDE | |  |  |
| Z4117 | | | *fucA* | L-fuculose-phosphate aldolase | | NDE |  | NDE |  | NDE | |  |  |
| Z4119 | | | *fucI* | L-fucose/D-arabinose isomerase | | NDE |  | 2.53 | 2.02E-03 | 3.65 | | 2.22E-06 |  |
| Z4120 | | | *fucK* | L-fuculokinase | | NDE |  | 2.01 | 7.21E-03 | 2.96 | | 2.30E-05 |  |
| Z4116 | | | *fucO* | Lactaldehyde reductase | | NDE |  | NDE |  | NDE | |  |  |
| Z4118 | | | *fucP* | L-fucose permease | | NDE |  | 2.52 | 8.56E-04 | 3.48 | | 1.10E-06 |  |
| Z4121  Z0462  Z0463 | | | *fucU*  *fusK*  *fusR* | L-fucose mutarotase  Fucose sensing two-component system (sensor)  Fucose sensing two-component system (regulator) | | NDE  NDE  NDE |  | NDE  NDE  2.09 | 5.02E-05 | 2.45  NDE  2.88 | | 7.21E-05  4.202E-07 |  |
| Z5632 | | | *malE* | Maltose/maltodextrin transport system (substrate-binding protein) | | NDE |  | NDE |  | NDE | |  |  |
| Z5631 | | | *malF* | Maltose/maltodextrin transport system (permease protein) | | NDE |  | NDE |  | NDE | |  |  |
| Z5630 | | | *malG* | Maltose/maltodextrin transport system (permease protein) | | NDE |  | NDE |  | NDE | |  |  |
| Z5633 | | | *malK* | Multiple sugar transport system (ATP-binding protein) | | 2.69 | 8.88E-05 | NDE |  | NDE | |  |  |
| Z3404 | | | *mglA* | Methyl-galactoside transport system (ATP-binding protein) | | NDE |  | NDE |  | 3.10 | | 3.25 E-05 |  |
| Z3405 | | | *mglB* | Methyl-galactoside transport system (substrate-binding protein) | | NDE |  | NDE |  | 3.05 | | 1.09 E-05 |  |
| Z3403 | | | *mglC* | Methyl-galactoside transport system (permease protein) | | NDE |  | NDE |  | NDE | |  |  |
| Z5250 | | | *rbsA* | Ribose transport system ATP-binding protein | | NDE |  | 2.95 | 1.83E-05 | NDE | |  |  |
| Z5252 | | | *rbsB* | Ribose transport system substrate-binding protein | | -2.06 | 0.02 | NDE |  | NDE | |  |  |
| Z5251 | | | *rbsC* | Ribose transport system permease protein | | NDE |  | 2.24 | 5.45E-04 | NDE | |  |  |
| Z5249 | | | *rbsD* | D-ribose pyranase | | NDE |  | 2.70 | 7.34E-05 | NDE | |  |  |
| Z5253 | | | *rbsK* | Ribokinase | | NDE |  | NDE |  | NDE | |  |  |
| Z4990 | | | *xylA* | Xylose isomerase | | 2.64 | 3.9E-05 | NDE |  | 2.56 | | 2.38E-05 |  |
| Z4989 | | | *xylB* | Xylulokinase | | NDE |  | NDE |  | 2.23 | | 1.21E-04 |  |
| Z4991 | | | *xylF* | D-xylose transport system (substrate-binding protein) | | 4.11 | 1.47E-06 | 2.29 | 8.40E-03 | 3.23 | | 7.53E-05 |  |
| Z4992 | | | *xylG* | D-xylose transport system (ATP-binding protein) | | 2.23 | 4.84E-04 | NDE |  | NDE | |  |  |
| Z4993  Z0461 | | | *xylH*  *z0461* | D-xylose transport system (permease protein)  Fucose tansporter (optimal import) | | NDE  NDE |  | NDE  2.42 | 4.55E-04 | NDE  3.59 | | 7.30E-06 |  |
| NDE: not differentially expressed | | | | | | | | | | | | | |
